# Associations between novel triglyceride glucose and adiposity-related indices and risk of metabolic dysfunction-associated steatotic liver disease: the mediating role of biological aging and evidence from the UK Biobank

**DOI:** 10.3389/fendo.2026.1915819

**Published:** 2026-08-11

**Authors:** Kun Wang, Renfang Han, Yueqing Huang, Jingfen Cai, Xiadi Wu

**Affiliations:** 1 Department of Science and Education, Wuxi Maternity and Child Health Care Hospital, Affiliated Women’s Hospital of Jiangnan University, Wuxi, China; 2 Department of Health Management Center, The Affiliated Suzhou Hospital of Nanjing Medical University, Suzhou Municipal Hospital, Suzhou, China; 3 Departments of General Medicine, The Affiliated Suzhou Hospital of Nanjing Medical University, Suzhou Municipal Hospital, Suzhou, China; 4 Department of Women' Health, Wuxi Maternity and Child Health Care Hospital, Affiliated Women’s Hospital of Jiangnan University, Wuxi, China

**Keywords:** adiposity, biological aging, cohort study, MASLD, triglyceride glucose-related index, UK Biobank

## Abstract

**Background:**

Insulin resistance (IR) plays a critical role in the development and prognosis of metabolic dysfunction-associated steatotic liver disease (MASLD). However, evidence regarding associations between novel triglyceride glucose (TyG) and adiposity-related composite indices, serving as simple IR surrogates, and the risk of incident MASLD remains limited. This study aimed to evaluate associations between six novel TyG-related composite indices and MASLD risk, as well as the potential mediating role of biological aging.

**Methods:**

This prospective cohort study included 426,831 participants from the UK Biobank, who were free of MASLD at baseline. Six novel TyG and adiposity-related composite indices [e.g., TyG-body mass index (BMI), TyG-waist circumference (WC), TyG-waist-to-height ratio (WHtR), TyG-body roundness index (BRI), TyG-a body shape index (ABSI), and TyG-weight-adjusted-waist index (WWI)] were calculated. Kaplan-Meier curves, Cox proportional hazards models, and restricted cubic splines (RCS) analyses were applied to evaluate associations between novel TyG-related indices and MASLD risk. Predictive performance was evaluated using Harrell’s C-index, net reclassification index (NRI), and integrated discrimination improvement index (IDI). Mediation analyses were performed to explore the potential role of biological aging. Additionally, MRI-measured proton density fat fraction (PDFF) was used to defined MASLD and investigate its association with TyG-related indices.

**Results:**

Over a median follow-up of 13.72 years, 6,308 new-onset MASLD cases were recorded. All six TyG-related composite indices were significantly associated with an increased risk of incident MASLD. Compared with the lowest quartile, hazard ratios (HRs) and 95% confidence intervals (CIs) for the highest quartile were 6.28 (5.62-7.02) for TyG-BMI, 8.27 (7.34-9.30) for TyG-WC, 8.18 (7.24-9.23) for TyG-WHtR, 7.79 (6.91-8.78) for TyG-BRI, 3.86 (3.49-4.26) for TyG-ABSI, and 6.06 (5.42-6.78) for TyG-WWI. RCS analyses revealed nonlinear relationships of all TyG-related indices and MASLD risk (all *P* for non-linearity <0.001). Incorporating novel TyG-related indices significantly improved predictive performance, with the greatest gains observed for TyG-WHtR and TyG-WC. Mediation analyses indicated that biological aging, assessed by KDM-BA and PhenoAge, accounted for 3%-12% of these associations. Results were consistent across subgroup and sensitivity analyses and when MASLD was defined as PDFF ≥5%.

**Conclusion:**

Six novel TyG and adiposity-related composite indices were independently associated with the risk of incident MASLD. These indices, particularly TyG-WHtR and TyG-WC, may server as simple and effective predictors for MASLD. Biological aging partially mediated these associations, offering novel insights into the pathways from IR to MASLD development.

## 1 Background

Non-alcoholic fatty liver disease (NAFLD) was renamed to metabolic dysfunction–associated steatotic liver disease (MASLD) in 2023, marking a shift from an exclusion-based definition to an inclusive, positive framework (1). This change not only provides a clear clinical diagnostic framework, but also reduces disease-related stigma. As the most common chronic liver disease, MASLD is characterized by excessive fat accumulation in the liver along with metabolic dysfunctions and has posed a major global public health challenge (1–3). MASLD affects 38% of population worldwide, and its prevalence is projected to rise substantially in the coming decades (4). Mounting evidence indicates that MASLD not only contributes to severe liver-related events, including advanced fibrosis, cirrhosis, and hepatocellular carcinoma, but also significantly heightens the risk of type 2 diabetes, cardiovascular disease (CVD), extrahepatic cancers, and premature death (5–8). Up to now, effective and targeted pharmacotherapies for MASLD are still rare (3, 9), therefore, identifying reliable and clinically accessible predictors of MASLD has become a public health and clinical priority.

The pathophysiology of MASLD is complex and several mechanisms interactively contribute to its onset and progression (2, 8). Insulin resistance (IR), defined as a diminished sensitivity and responsiveness of insulin in blood glucose utilization (10), is considered as the core pathological mechanism underlying MASLD development (1, 11). The gold-standard methods for assessing insulin include the hyperinsulinemic-euglycemic clamp and the homeostatic model assessment of IR (HOMA-IR) (12, 13). However, their invasiveness, expensive costs, and procedural complexity limit their widespread use in routine clinical practices and large epidemiological studies (13). Recently, triglyceride-glucose (TyG) index has been proposed as a simple surrogate marker for IR, with a comparable performance in evaluating IR to traditional approaches (14). Previous studies have shown that higher TyG levels were significantly associated with an increased risk of MASLD/NAFLD (11, 15, 16). Another study reported that persistently high TyG level was associated with increased risks of liver-related adverse outcomes (17). Besides, several novel indices combining TyG and adiposity measures, such as TyG-body mass index (BMI), TyG-waist circumference (WC), TyG-waist-to-height ratio (WHtR), TyG-body roundness index (BRI), TyG-a body shape index (ABSI), and TyG-weight-adjusted-waist index (WWI), have been shown to improve predictive performance for cardiometabolic conditions (18–22). Comparing with TyG index alone, these integrated indices could provide complementary pathophysiological information to identify individuals in whom IR coexists with specific high-risk patterns of fat accumulation (20). However, evidence on their associations with MASLD risk remains limited. To our knowledge, only one prior study has examined associations of these novel indices (including TyG-BMI, TyG-WC, and TyG-WHtR) with the risk of MASLD in Chinese adults (23), but it was constrained by small sample size, short follow-up period, insufficient adjustment of important confounders, and restriction to Chinese adults. And relationships between MASLD and other novel TyG-related indices (e.g., TyG-BRI, TyG-ABSI, and TyG-WWI) were still underexamined. Furthermore, magnetic resonance imaging (MRI)-measured liver proton density fat fraction (PDFF) offers a precise and reliable method to estimate liver fat content and serves as an effective tool for detecting hepatic steatosis (24), yet its associations with the aforementioned novel composite indices remain unclear.

Biological aging, a multidimensional indicator reflecting an individual’s overall physiological state, has emerged as a robust predictor of multiple morbidities and mortality (25, 26). Accumulating evidences demonstrate that biological aging significantly exacerbates the development and prognosis of MASLD, given its close association with aging-related metabolic dysregulation (27–30). Additionally, IR may further promote biological aging through chronic inflammation, oxidative stress, accelerating vascular aging, and worsening endothelial function (31, 32). Indeed, biological aging has been reported to mediate the associations of TyG and obesity-related indices with the risk of CVD and death (31, 33, 34). Thus, we hypothesize that biological aging may serve as a potential mediator linking the relationship between TyG-related indices and MASLD risk.

To fill these knowledge gaps, we conducted a prospective cohort study based on UK Biobank study to comprehensively investigate the associations between six novel TyG-related composite indices and the risk of incident MASLD, and to compare their predictive performance for MASLD. In addition, we explored the mediating role of biological aging in the associations between TyG-related indices and MASLD risk. Finally, based on objectively measured MRI-derived data, we further assessed associations between six novel TyG-related indices and PDFF-defined MASLD.

## 2 Methods

### 2.1 Data sources

Data used in this study were derived from the UK Biobank study, an ongoing population-based epidemiological study that recruited over 500,000 participants aged 40–69 years from the United Kingdom between 2006 and 2010 (35). At baseline, community residents were invited to undertake physical measurements, complete questionnaires, and participate in face-to-face interviews in one of 22 assessment centers across England, Scotland, and Wales. Ultimately, comprehensive information on demographic characteristics, socioeconomic background, lifestyle behaviors, health status, medication use, and disease history were collected. The UK Biobank study were approved by the Northwest Multicenter Research Ethics Committee (reference: 21/NW/0157) and all participants provided written informed consent before the baseline assessment.

### 2.2 Study population

**Figure 1:**
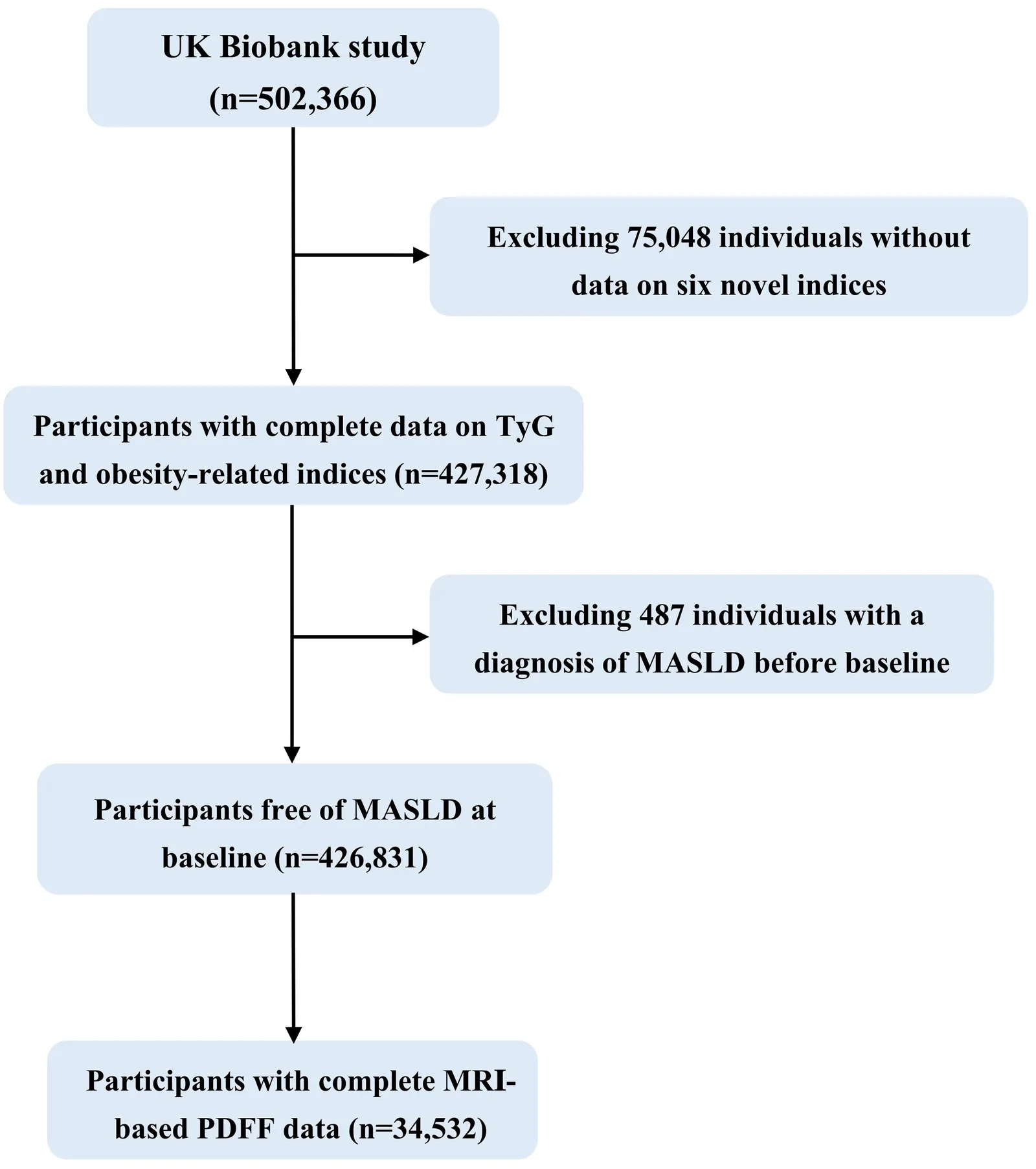
Flowchart of the inclusion and exclusion of study population. TyG triglycerides glucose index; MASLD metabolic dysfunction-associated steatotic liver disease; MRI magnetic resonance imaging; PDFF liver proton density fat fraction.

The present study initially excluded individuals without complete data on novel TyG-related indices (n=75,048) and those with a diagnosis of MASLD prior to baseline assessment (n=487). A total of 426,831 participants were included to investigate the associations of novel TyG-related composite indices with risk of incident MASLD. Of 426,831 participants, we included 34,532 participants with available MRI data to explore the associations between TyG-related indices and the presence of MASLD defined on the basis of MRI-measured PDFF. **Figure 1** shows the detailed procedure of population selection.

### 2.3 Definition of exposure variables

Participants provided the random peripheral blood samples at baseline survey and biochemical assays were conducted using the Beckman Coulter AU5800 chemistry analyzer (36). Detailed information on collection, processing, and quality control can be found on the UK Biobank website (https://biobank.ndph.ox.ac.uk/showcase/refer.cgi?id=1227). Briefly, triglycerides and glucose were measured using GPO-POD analysis and hexokinase analysis, respectively. Weight, height, and WC for each participant were measured by well-trained staff. In the current study, six novel TyG and adiposity-related novel indices that integrate the TyG index and anthropometric parameters, including TyG-BMI, TyG-WC, TyG-WHtR, TyG-BRI, TyG-ABSI, and TyG-WWI, were calculated using the following formulas (18, 22, 33, 37):


$$\text{TyG}-\text{BMI}=TyG\times weight(kg)/height^{2}(m^{2})$$

TyG−WC=TyG×WC(cm)

TyG−WHtR=TyG×[WC(cm)/height(cm)]

$$\text{TyG}-\text{BRI}=TyG\times 364.2-365.5\times \sqrt{1-{\left(\frac{WC(cm)}{2\pi }\right)}^{2}/{\left(\frac{height(cm)}{2}\right)}^{2}}$$

$$\text{TyG}-\text{ABSI}=TyG\times WC(cm)/BMI{\left(kg/m^{2}\right)}^{2/3}\times height{\left(m\right)}^{1/2})$$

$$\text{TyG}-\text{WWI}=TyG\times \text{WC}(\text{cm})/\sqrt{weight(kg)}$$

TyG=ln[TG(mg/dL)×blood glucose(mg/dL)/2]

### 2.4 Assessment of outcome

Primary outcomes in the present study were the occurrence of new-onset MASLD and the presence of PDFF-defined MASLD. In the UK Biobank study, new-onset MASLD cases were identified through electronic medical record in the inpatient admission records and death registry data. Incident events were ascertained based on the International Classification of Diseases, 10th Revision (ICD-10) codes, specifically K76.0 and K75.8 for MASLD (38). Mortality date and cause data were obtained from death certificates from the National Health Service Information Centre for England and Wales, and the Information and Statistics Division for Scotland. The follow-up period was calculated from the date of baseline enrollment to the date of MASLD occurrence, death, or November 30, 2022, whichever occurred first. Besides, we used liver PDFF assessed using MRI scans to additionally define MASLD. Approximately 4 years after the baseline survey, 84,000 participants were invited to undertake abdominal MRI measurement using a Siemens 1.5T MAGNETOM Aera scanner (Siemens Healthineers, Erlangen, Germany) with a 6 min dual‐echo Dixon Vibe protocol (39). Liver MRI data were analysed using Liver*Multi*Scan™ Discover software, and PDFF was calculated using the formulas: PDFF = fat/(fat + water). Detailed information on MRI measurement can be found at https://biobank.ndph.ox.ac.uk/showcase/refer.cgi?id=348. According to previous study (40, 41), we define MASLD as PDFF ≥5%.

### 2.5 Measurement of biological aging

Biological aging was assessed via two validated algorithms that could be measured with available data in the UK Biobank: Klemera-Doubal method Biological Age (KDM-BA) and the PhenoAge algorithms (42–44). KDM-BA was calculated using forced expiratory volume in one second (FEV_1_), systolic blood pressure (SBP), albumin, alkaline phosphatase, blood urea nitrogen, creatinine, C-reactive protein, glycated hemoglobin, and total cholesterol (TC); PhenoAge was derived from nine blood chemistries biomarkers, including albumin, alkaline phosphatase, creatinine, C-reactive protein, blood glucose, mean cell volume, red cell distribution width, white blood cell count, and lymphocyte proportion (42). Detailed information on the included biomarkers and corresponding data fields in UK Biobank was provided in **Supplementary Table 1**. Both biological aging measurements were calculated using the “BioAge” package in R statistic software (45).

### 2.6 Covariates

According to prior related studies and clinical knowledge (15, 23), potential covariates included age (continuous variable, year), sex, race (categorical variable, White *vs*. non-White), employment status (categorical variable, yes *vs*. no), Townsend deprivation index (TDI) (continuous variable, with higher scores indicating greater deprivation), total family income (categorical variable, low *vs*. middle *vs*. high). Lifestyle factors included smoking status, alcoholic drinking, physical activity, diet, sleep duration. Smoking status were categorized into never, former, and current smoking, and alcoholic drinking was classified into never, <3 times/week, ≥3 times/week. Physical activity was defined adequate according to WHO recommendations (e.g., ≥150 minutes/week of moderate-to-vigorous intensity physical activity) and inadequate. A healthy diet score was conducted using seven dietary components, including fruit, vegetable, fish, whole grains, refined grains, processed meats, and unprocessed meats, with higher scores indicating healthier die. Sleep duration was categorized into <7 hours/day, 7–8 hours/day, and >8 hours/day. Clinical variables included TC (continuous variable, mmol/L), low-density lipoprotein cholesterol (LDL-C) (continuous variable, mmol/L), SBP and diastolic blood pressure (DBP) (continuous variable, mmHg), use of antihypertensive, antihyperglycemic, and lipid-lowering medications (categorical variable, yes *vs*. no), and self-reported history of hypertension, diabetes, and hyperlipidemia (categorical variable, yes *vs*. no). To maximize sample size, we assigned an additional categorical category “missing” for missing categorical covariates and imputed median values for missing continuous covariates, consistent with common practices in previous studies (46, 47).

### 2.7 Statistical analysis

Baseline characteristics of the study population were summarized stratified by the occurrence of MASLD during follow-up and quartiles of novel TyG-related composite indices. Continuous variables were presented as means and standard deviation (SD) for normally distributed variables and median with interquartile ranges (IQR) for skewed variables. Categorical variables were expressed as count and percentages (%). Group differences were compared using *t* tests, one-way analysis of variance (ANOVA), Keuskal-Wallis tests, or chis-square (*χ*^2^) test, as appropriate. Additionally, we also described the baseline characteristics of participants with available PDFF data and compared according to the presence of MASLD, defined as PDFF ≥5%.

We first investigated the prospective associations between six novel TyG-related indices and the risk of incident MASLD. Kaplan-Meier curves were applied to visually compare the cumulative hazard of MASLD across quartiles of TyG-related indices, with log-rank test for statistical assessment. Cox proportional hazard regression models were used to estimate hazard ratio (HR) and 95% confidence interval (CI). Schoenfeld residuals test was utilized to evaluate the proportional hazard assumption, and no violations were observed. Exposures were analyzed as per 1-SD increase (continuous variable) and by quartiles (Q2-Q4), with the lowest quartile (Q1) as the reference (categorical variable). Three modes with incremental adjustment for covariates were established: Model 1 adjusted for age and sex, Model 2 additionally adjusted for race, employed status, TDI, total family income, smoking status, drinking frequency, healthy diet score, physical activity, and sleep duration, and Model 3 further adjusted for TC, HbA1c, SBP, DBP, use of antihypertensive, antihyperglycemic, and lowering-lipid medications, and self-reported history of diabetes, hypertension, and hyperlipidemia. To assess whether there was a potential non-linear relationship between TyG-related indices and future risk of developing MASLD, restricted cubic splines (RCS) with four knots placed at 5th, 35th, 65th, and 95th percentiles were fitted within the Cox proportional hazard regression framework.

Mediation analyses were performed to evaluate the potential contribution of biological aging, assessed by KDM-BA and PhenoAge, in the associations between TyG-related indices and MALSD risk. First, multiple linear regression models were used to investigate the associations of six novel TyG-related indices with biological aging measures (e.g., KDM-BA and PhenoAge). Results were reported as regression coefficient (*β*) with 95% CI per 1-SD increase. Second, Cox proportional hazard regression models were used to performed to investigate the association of biological aging measurements with MASLD risk. Final, causal mediation analyses by “CMAverse” package were conducted to assess whether biological aging mediated the association between TyG-related indices and MASLD risk and to estimate the mediation proportions (48).

Furthermore, we examine the associations between six novel TyG-related indices and the odds of prevalent PDFF-defined MASLD. Logistical regression models were used to estimate odd ratio (OR) and 95% CI. Three models were also conducted using the same set of confounders in Cox proportional hazard regression models. Similarly, RCS analyses within logistic regression models were utilized to evaluate the potential non-linear relationships.

A series of additional analyses were performed to assess the robustness of our results. First, primary analyses were repeated after imputing the missing covariate data using the multiple imputation by chained equations (MICE) to reduce the potential influence of missing covariates data. Second, to mitigate the reverse causation, participants who occurred MASLD within the first 2 years of follow-up were excluded. Third, given that individuals with other liver diseases were at significantly elevated risk of CVD and mortality, associations were reanalyzed after excluding those with other liver diseases before/at baseline. Fourth, the definition of MASLD outcome was restricted to K76.0 to evaluate the robustness of results. Final, subgroup analyses stratified by age (<60 *vs*. ≥60 years), sex (female *vs*. male), smoking status (non-smoker *vs*. ever or current smoker), alcoholic drinking (non-drinker *vs*. drinker), self-reported history of hypertension, diabetes, and hyperlipidemia (yes *vs*. no) were performed to explore the heterogeneity population. Modification effects of stratified variables were examined using likelihood ratio tests.

## 3 Results

### 3.1 Baseline characteristics of participants

Baseline characteristics of the study population both overall and stratified by the occurrence of MASLD were presented in **Table 1**, while characteristics according to quartiles of TyG-BMI were shown in **Supplementary Table 2**. Of the 426,831 participants, mean age at baseline was 56.55 (SD: 8.09) years old, 222,933 (53.87%) were women, and 402,586 (94.32%) were Whites. Compared with participants who did not develop MASLD, those with MASLD occurrence were more likely to be older, male, non-White, unemployed, without a university or college degree, and more socioeconomically deprived. They also exhibited a higher prevalence of inadequate physical activity, ever or current smoking, inappropriate daily sleep duration, unhealthy diet, use of antihypertensive, antihyperglycemic, and lipid-lowering medications, and a self-reported history of diabetes, hypertension, and dyslipidemia. Moreover, levels of six novel TyG-related indices, TC, LDL-C, HbA1C, SBP, and DBP were significantly higher among participants who developed MASLD. Similar patterns in baseline characteristics were observed across quartiles of TyG-BMI (**Supplementary Table 2**).

**Table 1:** Baseline characteristics of study population stratified by MASLD occurrence.

Characteristics	Overall	MASLD incidence		*P* value
		Yes	No	
n	426,831	6,308	420,523	
Age, year	56.55 ± 8.09	56.98 ± 7.88	56.54 ± 8.09	<0.001
Female	229,933 (53.87)	3,238 (51.33)	226,695 (53.91)	<0.001
White	402,586 (94.32)	5,872 (93.09)	396,714 (94.34)	<0.001
Employed status				<0.001
Employed	390001 (91.37)	5318 (84.31)	384683 (91.48)	
Unemployed	34699 (8.13)	953 (15.11)	33746 (8.02)	
Missing	2131 (0.50)	37 (0.59)	2094 (0.50)	
Educational levels				<0.001
University or college	137943 (32.32)	1370 (21.72)	136573 (32.48)	
Otders	283852 (66.50)	4832 (76.60)	279020 (66.35)	
Missing	5036 (1.18)	106 (1.68)	4930 (1.17)	
Townsend deprivation index	-2.15 (-3.65, 0.51)	-1.15 (-3.14, 2.15)	-2.16 (-3.66, 0.48)	<0.001
Family income				<0.001
Low	82773 (19.39)	1746 (27.68)	81027 (19.27)	
Intermediate	187866 (44.01)	2582 (40.93)	185284 (44.06)	
High	94100 (22.05)	887 (14.06)	93213 (22.17)	
Missing	62092 (14.55)	1093 (17.33)	60999 (14.51)	
Physical activity				<0.001
Inadequate	73242 (17.16)	1308 (20.74)	71934 (17.11)	
Adequate	291333 (68.25)	3585 (56.83)	287748 (68.43)	
Missing	62256 (14.59)	1415 (22.43)	60841 (14.47)	
Smoking status				<0.001
Never smoking	232213 (54.40)	2805 (44.47)	229408 (54.55)	
Ever smoking	147575 (34.57)	2523 (40.00)	145052 (34.49)	
Current smoking	44920 (10.52)	940 (14.90)	43980 (10.46)	
Missing	2123 (0.50)	40 (0.63)	2083 (0.50)	
Alcoholic drinking				<0.001
Never	34033 (7.97)	822 (13.03)	33211 (7.90)	
Less tdan tdree times/montd	206281 (48.33)	3249 (51.51)	203032 (48.28)	
tdree times/week or more	185621 (43.49)	2211 (35.05)	183410 (43.61)	
Missing	896 (0.21)	26 (0.41)	870 (0.21)	
Sleep duration				<0.001
<7 hours/day	104566 (24.50)	1926 (30.53)	102640 (24.41)	
7–9 hours/day	311423 (72.96)	4040 (64.05)	307383 (73.10)	
>9 hours/day	7705 (1.81)	246 (3.90)	7459 (1.77)	
Missing	3137 (0.73)	96 (1.52)	3041 (0.72)	
Healtdy diet score	3.31 ± 1.32	3.12 ± 1.33	3.31 ± 1.32	<0.001
TyG-BMI index	233.42 (204.72, 267.49)	276.40 (243.27, 316.19)	232.87 (204.39, 266.67)	<0.001
TyG-WC index	790.62 ± 147.65	910.71 ± 153.52	788.82 ± 146.82	<0.001
TyG-WHtR index	4.69 ± 0.83	5.43 ± 0.88	4.68 ± 0.83	<0.001
TyG-BRI index	34.51 (25.88, 44.70)	47.62 (37.83, 59.95)	34.34 (25.77, 44.45)	<0.001
TyG-ABSI index	6.69 ± 0.74	7.10 ± 0.73	6.68 ± 0.73	<0.001
TyG-WWI index	89.41 ± 10.47	97.09 ± 10.41	89.30 ± 10.42	<0.001
TC, mmol/L	5.69 ± 1.14	5.49 ± 1.26	5.70 ± 1.14	<0.001
LDL-C, mmol/L	3.56 ± 0.87	3.44 ± 0.94	3.56 ± 0.87	<0.001
HbA1C, %	36.06 ± 6.55	39.25 ± 10.09	36.01 ± 6.47	<0.001
SBP, mmHg	137.95 ± 18.63	140.60 ± 18.12	137.91 ± 18.64	<0.001
DBP, mmHg	82.30 ± 10.14	84.51 ± 10.17	82.27 ± 10.14	<0.001
Use of medications				
Antihypertensive medication	88860 (20.82)	2321 (36.79)	86539 (20.58)	<0.001
Antihyperglycemic medication	15927 (3.73)	807 (12.79)	15120 (3.60)	<0.001
Lipid-lowering medication	74313 (17.41)	1986 (31.48)	72327 (17.20)	<0.001
Self-reported disease history				
Hypertension	117279 (27.48)	2867 (45.45)	114412 (27.21)	<0.001
Diabetes	22304 (5.23)	1072 (16.99)	21232 (5.05)	<0.001
Hyperlipidemia	52818 (12.37)	1303 (20.66)	51515 (12.25)	<0.001

Data were described as mean ± standard deviation (SD) for normally-distributed continue variables and median and interquartile ranges (IQR) for skewedly distributed continue variables, and categorical variables were described as number and precent (%). Missing covariate data were imputed using additional category “Missing” for categorical variables and median values for continue variables.

MASLD metabolic dysfunction-associated steatotic liver disease; TyG triglycerides glucose index, BMI body mass index, WC waist circumference, WHtR waist-to-height ratio; BRI body roundness index; ABSI a body shape index; WWI weight-adjusted-waist index, TC total cholesterol; LDL-C low density lipoprotein cholesterol; HbA1C glycated hemoglobin, SBP systolic blood pressure; DBP diastolic blood pressure.

**Supplementary Table 3** presented the baseline characteristics of 34,532 participants with complete PDFF data, stratified by the prevalence of MASLD defined by PDFF ≥5%. The mean age was 55.04 (SD: 7.52) years, 17,881 (51.78%) were females, and 33,411 (96.75%) were Whites. Compared with individuals without MASLD, those with PDFF-defined MASLD tended to be older, male, less educated, socioeconomically deprived, smoker, and have lower family income. They also tended to consume less alcohol, have inappropriate sleep duration, and follow a poorer diet. Besides, individuals with MASLD exhibited higher levels of TyG-BMI, TyG-WC, TyG-WHtR, TyG-BRI, TyG-ABSI, TyG-WWI, TC, HbA1C, SBP, and SBP, and have a higher prevalence of antihypertensive, antihyperglycemic, and lipid-lowering medication use, as well as a greater disease history.

### 3.2 Associations of novel TyG-related indices with MASLD incidence

**Figure 2:**
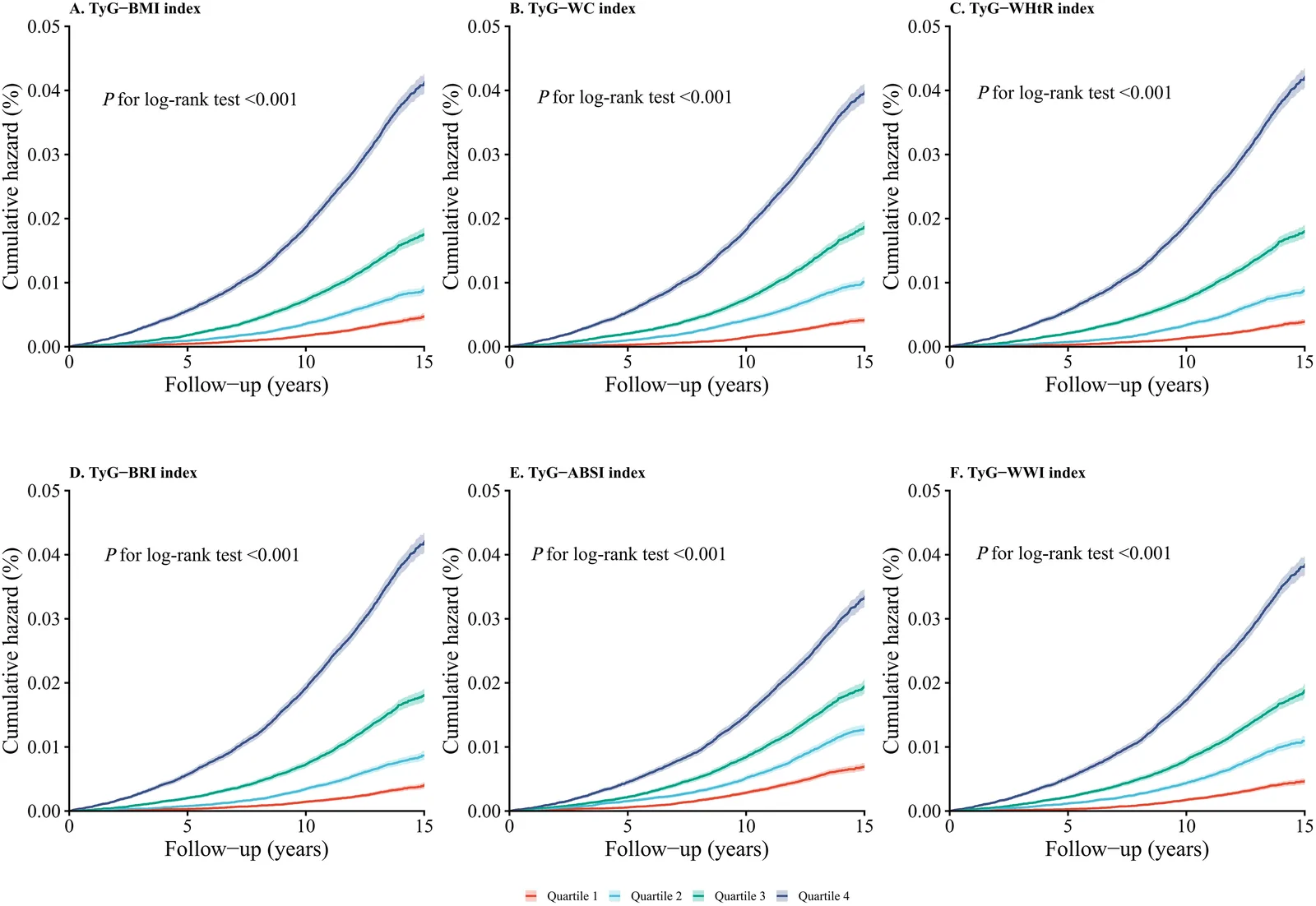
Kaplan-Meier curves of incident MASLD stratified by quartiles of six novel TyG and adiposity-related indices (**(A)** TyG-BMI, **(B)** TyG-WC, **(C)** TyG-WHtR, **(D)** TyG-BRI, **(E)** TyG-ABSI, **(F)** TyG-WWI). MASLD metabolic dysfunction-associated steatotic liver disease; TyG triglycerides glucose index; BMI body mass index, WC waist circumference, WHtR waist-to-height ratio; BRI body roundness index; ABSI a body shape index; WWI weight-adjusted-waist index.

**Figure 3:**
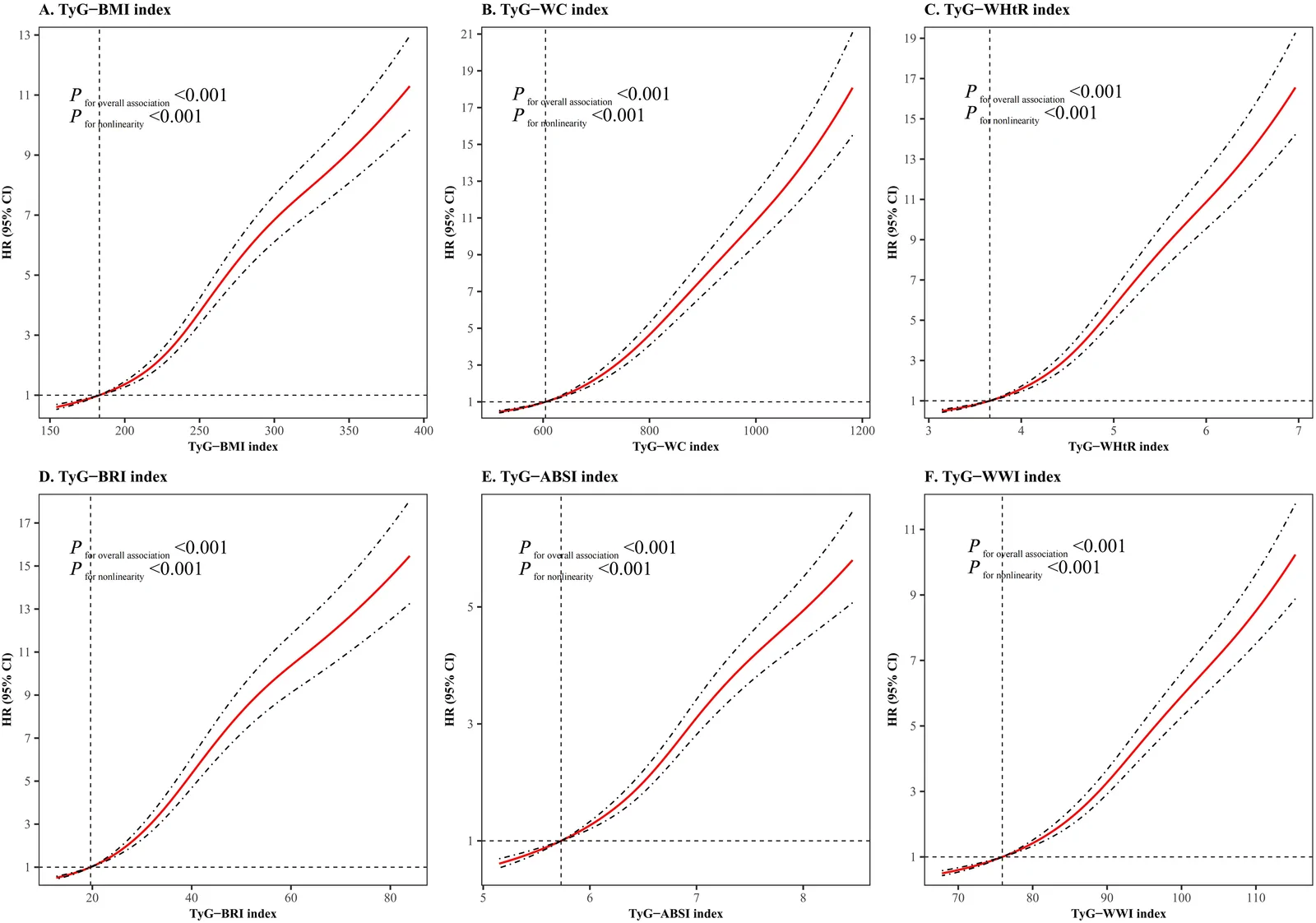
Restricted cubic spline analyses of six novel TyG and adiposity-related indices in relation to incident MASLD risk **(A)** TyG-BMI index, **(B)** TyG-WC index, **(C)** TyG-WHtR index, **(D)** TyG-BRI index, **(E)** TyG-ABSI index, **(F)** TyG-WWI index). Models were adjusted for age, sex, ethnicity, employed status, educational levels, Townsend deprivation index, family income, physical activity, smoking status, alcoholic drinking, sleeping duration, health diet score, total cholesterol, low density lipoprotein cholesterol, glycated hemoglobin, systolic/diastolic blood pressure, use of antihypertensive, antihyperglycemic, lipid-lowering medications, and self-reported history of hypertension, diabetes, and hyperlipidemia. HR hazard ratio, CI confidence interval, MASLD metabolic dysfunction-associated steatotic liver disease; TyG triglycerides glucose index, BMI body mass index, WC waist circumference, WHtR waist-to-height ratio; BRI body roundness index; ABSI a body shape index; WWI weight-adjusted-waist index.

During a median follow-up of 13.72 (IQR: 12.95-14.44) years, a total of 6,308 new-onset MASLD cases were identified, corresponding to an incident rate of 11.09 per 10,000 person-years. Kaplan-Meier curves demonstrated a graded increase in the cumulative hazard of incident MASLD across higher quartiles of novel TyG-related indices (all *P* values for log-rank test <0.001, **Figures 2A–F**). After full adjustment for potential confounders, HRs of developing MASLD for per 1-SD increase in TyG-related indices were 1.58 (95% CI: 1.55-1.62) for TyG-BMI, 1.83 (95% CI: 1.79-1.88) for TyG-WC, 1.79 (95% CI: 1.74-1.83) for TyG-WHtR, 1.55 (95% CI: 1.52-1.58) for TyG-BRI, 1.59 (95% CI: 1.54-1.63) for TyG-ABSI, and 1.75 (95% CI: 1.70-1.80) for TyG-WWI, respectively (**Table 2**). In categorical analyses, risk of developing MASLD was significantly increased in participants in the higher quartiles (Q2-Q4) group than those in the first quartile group. Specifically, fully adjusted HRs for the highest quartiles (Q4) versus the lowest quartile (Q1) were 6.28 (5.62-7.02) for TyG-BMI, 8.27 (7.34-9.30) for TyG-WC, 8.18 (7.24-9.23) for TyG-WHtR, 7.79 (6.91-8.78) for TyG-BRI, 3.86 (3.49-4.26) for TyG-ABSI, and 6.06 (5.42-6.78) for TyG-WWI, respectively (**Table 2**). Furthermore, we evaluated the dose-response relationship of TyG-related indices and risk of MASLD. As shown in **Figure 3**, multivariable-adjusted RCS analyses revealed a non-linear, J-shaped relationship of TyG-BMI, TyG-WC, TyG-WHtR, TyG-BRI, TyG-ABSI, and TyG-WWI with risk of MASLD (all *P* values for non-linearity <0.001, **Figures 3A–F**).

**Table 2:** Association between novel triglyceride glucose-related indices and risk of metabolic dysfunction-associated steatotic liver disease.

Exposure	Cases/incidence rate	Model 1		Model 2		Model 3	
		HR (95% CI)	*P* value	HR (95% CI)	*P* value	HR (95% CI)	*P* value
TyG-BMI index							
Per 1-SD increase		1.86 (1.83-1.89)	<0.001	1.73 (1.69-1.76)	<0.001	1.58 (1.55-1.62)	<0.001
Quartile 1	399/0.28	Reference	–	Reference	–	Reference	–
Quartile 2	780/0.55	1.97 (1.75-2.23)	<0.001	1.91 (1.69-2.15)	<0.001	1.87 (1.66-2.12)	<0.001
Quartile 3	1535/1.08	3.93 (3.52-4.39)	<0.001	3.58 (3.20-4.00)	<0.001	3.37 (3.01-3.78)	<0.001
Quartile 4	3594/2.57	9.41 (8.48-10.44)	<0.001	7.68 (6.91-8.53)	<0.001	6.28 (5.62-7.02)	<0.001
* P* for trend		<0.001		<0.001		<0.001	
TyG-WC index							
Per 1-SD increase		2.18 (2.13-2.23)	<0.001	2.00 (1.95-2.04)	<0.001	1.83 (1.79-1.88)	<0.001
Quartile 1	370/0.26	Reference	–	Reference	–	Reference	–
Quartile 2	890/0.62	2.77 (2.45-3.13)	<0.001	2.56 (2.27-2.89)	<0.001	2.51 (2.22-2.84)	<0.001
Quartile 3	1613/1.14	5.65 (5.04-6.34)	<0.001	4.90 (4.36-5.50)	<0.001	4.57 (4.05-5.14)	<0.001
Quartile 4	3435/2.47	13.22 (11.83-14.78)	<0.001	10.24 (9.15-11.45)	<0.001	8.27 (7.34-9.30)	<0.001
* P* for trend		<0.001		<0.001		<0.001	
TyG-WHtR index							
Per 1-SD increase		2.10 (2.06-2.14)	<0.001	1.93 (1.89-1.97)	<0.001	1.79 (1.74-1.83)	<0.001
Quartile 1	334/0.23	Reference	–	Reference	–	Reference	–
Quartile 2	779/0.54	2.50 (2.19-2.84)	<0.001	2.35 (2.07-2.68)	<0.001	2.34 (2.06-2.67)	<0.001
Quartile 3	1579/1.11	5.27 (4.67-5.93)	<0.001	4.65 (4.12-5.24)	<0.001	4.46 (3.95-5.05)	<0.001
Quartile 4	3616/2.6	12.44 (11.10-13.94)	<0.001	9.78 (8.71-10.97)	<0.001	8.18 (7.24-9.23)	<0.001
* P* for trend		<0.001		<0.001		<0.001	
TyG-BRI index							
Per 1-SD increase		1.81 (1.78-1.83)	<0.001	1.69 (1.66-1.72)	<0.001	1.55 (1.52-1.58)	<0.001
Quartile 1	339/0.23	Reference	–	Reference	–	Reference	–
Quartile 2	765/0.53	2.39 (2.11-2.72)	<0.001	2.27 (2.00-2.58)	<0.001	2.24 (1.97-2.55)	<0.001
Quartile 3	1593/1.12	5.18 (4.60-5.83)	<0.001	4.61 (4.09-5.19)	<0.001	4.33 (3.84-4.90)	<0.001
Quartile 4	3611/2.6	12.08 (10.79-13.53)	<0.001	9.53 (8.50-10.69)	<0.001	7.79 (6.91-8.78)	<0.001
* P* for trend		<0.001		<0.001		<0.001	
TyG-ABSI index							
Per 1-SD increase	\	1.92 (1.87-1.97)	<0.001	1.73 (1.68-1.77)	<0.001	1.59 (1.54-1.63)	<0.001
Quartile 1	632/0.44	Reference	–	Reference	–	Reference	–
Quartile 2	1140/0.8	2.04 (1.85-2.25)	<0.001	1.86 (1.68-2.05)	<0.001	1.75 (1.58-1.93)	<0.001
Quartile 3	1708/1.21	3.41 (3.10-3.75)	<0.001	2.92 (2.65-3.21)	<0.001	2.58 (2.34-2.85)	<0.001
Quartile 4	2828/2.04	6.29 (5.73-6.90)	<0.001	4.85 (4.42-5.33)	<0.001	3.86 (3.49-4.26)	<0.001
* P* for trend		<0.001		<0.001		<0.001	
TyG-WWI index							
Per 1-SD increase		2.08 (2.03-2.13)	<0.001	1.88 (1.84-1.93)	<0.001	1.75 (1.70-1.80)	<0.001
Quartile 1	414/0.29	Reference	–	Reference	–	Reference	–
Quartile 2	979/0.68	2.60 (2.31-2.91)	<0.001	2.38 (2.12-2.67)	<0.001	2.30 (2.04-2.58)	<0.001
Quartile 3	1632/1.15	4.59 (4.11-5.12)	<0.001	3.94 (3.53-4.40)	<0.001	3.63 (3.24-4.07)	<0.001
Quartile 4	3283/2.36	9.74 (8.77-10.83)	<0.001	7.43 (6.68-8.27)	<0.001	6.06 (5.42-6.78)	<0.001
* P* for trend		<0.001		<0.001		<0.001	

Model 1 were adjusted for age and sex.

Model 2 were adjusted for age, sex, ethnicity, employed status, educational levels, Townsend deprivation index, family income, physical activity, smoking status, alcoholic drinking, sleeping duration, and health diet score.

Model 3 were adjusted for age, sex, ethnicity, employed status, educational levels, Townsend deprivation index, family income, physical activity, smoking status, alcoholic drinking, sleeping duration, health diet score, total cholesterol, low density lipoprotein cholesterol, glycated hemoglobin, systolic/diastolic blood pressure, use of antihypertensive, antihyperglycemic, lipid-lowering medications, and self-reported history of hypertension, diabetes, and hyperlipidemia.

HR hazard ratio, CI confidence interval, SD standard deviation; TyG triglycerides glucose index, BMI body mass index, WC waist circumference, WHtR waist-to-height ratio; BRI body roundness index; ABSI a body shape index; WWI weight-adjusted-waist index.

### 3.3 Predictive performance

To evaluate the incremental predictive value of six novel TyG-related indices beyond traditional risk factors, we compared the conventional model (Model 3) with models additionally incorporating each novel TyG-related index. As shown in **Table 3**, adding TyG-related indices significantly improved the NRI, IDI, and C index for the risk of incident MASLD (all *P* < 0.001), with the greatest improvement observed for TyG-WHtR and TyG-WC. Compared with the basic model, the IDI values for new models were 0.804 (95% CI: 0.709-0.904) for TyG-WHtR, 0.787 (0.689-0.886) for TyG-WC, 0.628 (0.549-0.722) for TyG-BMI, 0.660 (0.572-0.763) for TyG-BRI, 0.599 (0.527-0.673) for TyG-WWI, and 0.366 (0.311-0.431) for TyG-ABSI, respectively (**Table 3**). The corresponding NRI values were 24.513 (23.431-25.454), 23.951 (22.948-24.975), 22.825 (21.526-24.107), 22.089 (20.833-23.075), 21.211 (19.744-22.253), and 16.869 (15.542-18.121), respectively (**Table 3**). For the C index, the values were 0.753 (0.747-0.759) for basic model, 0.756 (0.751-0.762) for TyG-WHtR, 0.759 (0.753-0.764) for TyG-WC, 0.753 (0.747-0.759) for TyG-BMI, 0.754 (0.748-0.760) for TyG-BRI, 0.743 (0.737-0.749) for TyG-WWI, and 0.727 (0.721-0.733) for TyG-ABSI, respectively (**Table 3**). These results indicate that incorporating novel TyG-related indices, particularly TyG-WHtR and TyG-WC, enhances the predictive accuracy of traditional risk models for MASLD.

**Table 3:** Increment predictive values of six novel triglyceride glucose-related indices for the risk of metabolic dysfunction-associated steatotic liver disease.

Exposure	IDI (%)	*P* value	NRI (%)	*P* value	C index	*P* value
Basic model	Reference		Reference		0.700 (0.694-0.707)	
+TyG-BMI index	0.628 (0.549-0.722)	<0.001	22.825 (21.526-24.107)	<0.001	0.753 (0.747-0.759)	<0.001
+TyG-WC index	0.787 (0.689-0.886)	<0.001	23.951 (22.948-24.975)	<0.001	0.756 (0.751-0.762)	<0.001
+TyG-WHtR index	0.804 (0.709-0.904)	<0.001	24.513 (23.431-25.454)	<0.001	0.759 (0.753-0.764)	<0.001
+TyG-BRI index	0.660 (0.572-0.763)	<0.001	22.089 (20.833-23.075)	<0.001	0.754 (0.748-0.760)	<0.001
+TyG-ABSI index	0.366 (0.311-0.431)	<0.001	16.869 (15.542-18.121)	<0.001	0.727 (0.721-0.733)	<0.001
+TyG-WWI index	0.599 (0.527-0.673)	<0.001	21.211 (19.744-22.253)	<0.001	0.743 (0.737-0.749)	<0.001

Comparisons were performed between basic models, which were conducted by including covariates in Model 3, including age, sex, ethnicity, employed status, educational levels, Townsend deprivation index, family income, physical activity, smoking status, alcoholic drinking, sleeping duration, health diet score, total cholesterol, low density lipoprotein cholesterol, glycated hemoglobin, systolic/diastolic blood pressure, use of antihypertensive, antihyperglycemic, lipid-lowering medications, and self-reported history of hypertension, diabetes, and hyperlipidemia, and new models, which incorporating TyG-related indices into basic models.

IDI integrated discrimination improvement, NRI net reclassification index; TyG triglycerides glucose index, BMI body mass index, WC waist circumference, WHtR waist-to-height ratio; BRI body roundness index; ABSI a body shape index; WWI weight-adjusted-waist index.

### 3.4 Mediation analyses of biological aging

**Figure 4:**
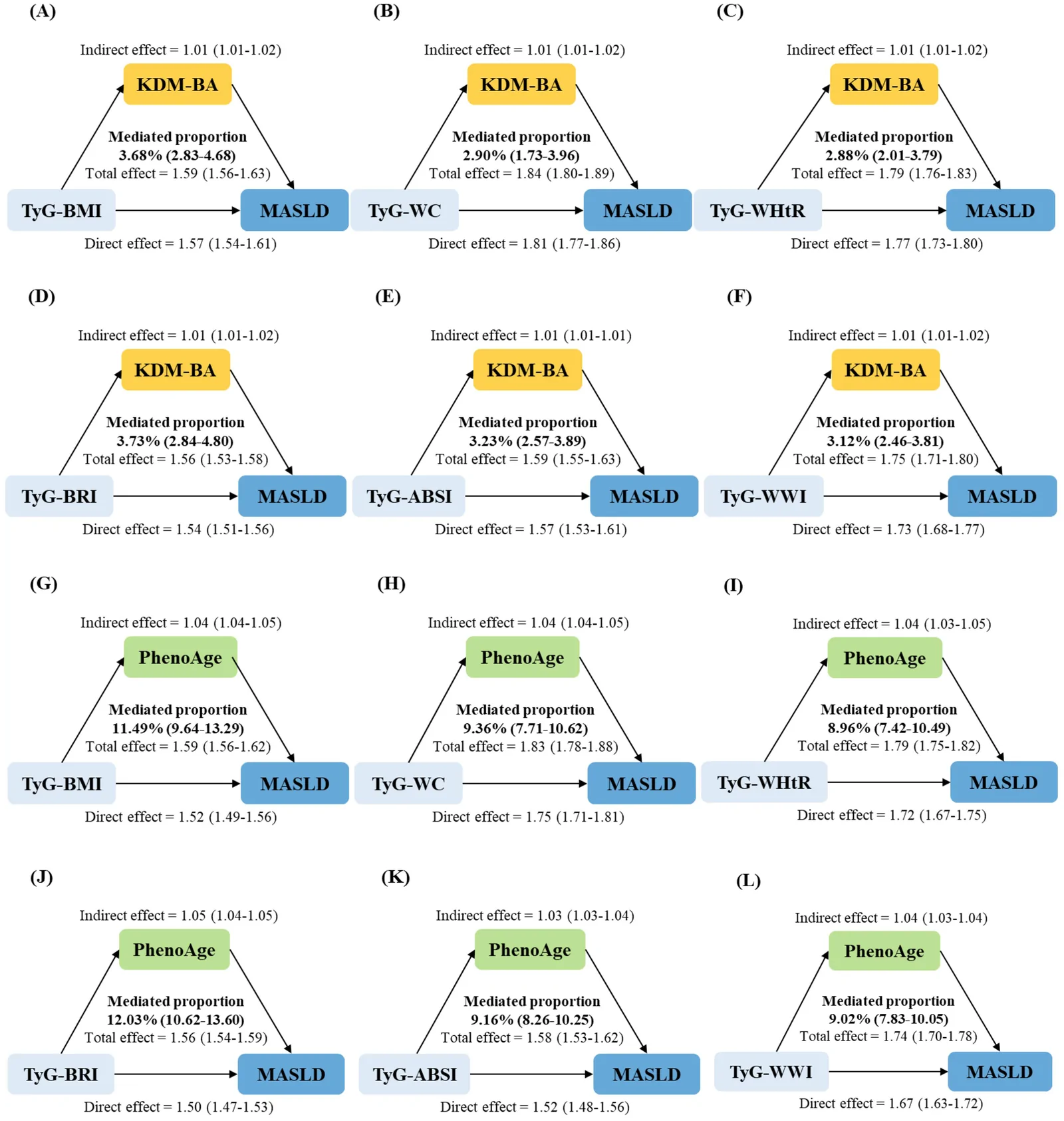
Mediated role of biological aging in the associations between novel TyG and adiposity-related indices and the risk of incident MASLD **(A–F)** for KDM-BA and **(G–L)** for PhenoAge. Biological aging was assessed by KDM-BA and PhenoAge. Mediation analyses were adjusted for age, sex, ethnicity, employed status, educational levels, Townsend deprivation index, family income, physical activity, smoking status, alcoholic drinking, sleeping duration, health diet score, total cholesterol, low density lipoprotein cholesterol, glycated hemoglobin, systolic/diastolic blood pressure, use of antihypertensive, antihyperglycemic, lipid-lowering medications, and self-reported history of hypertension, diabetes, and hyperlipidemia. MASLD metabolic dysfunction-associated steatotic liver disease; KDM-BA Klemera-Doubal method Biological Age; TyG triglycerides glucose index, BMI body mass index, WC waist circumference, WHtR waist-to-height ratio; BRI body roundness index; ABSI a body shape index; WWI weight-adjusted-waist index.

To explore the potential mediating role of biological aging in the association between TyG-related indices and risk of MASLD, mediation analyses were conducted. As shown in **Supplementary Table 4**, all six TyG-related indices were significantly associated with both KDM-BA and PhenoAge. Specifically, adjusted regression coefficients for per 1-SD increase in TyG-related indices ranged from 0.30 to 0.61 for KDM-BA and from 1.07 to 1.62 for PhenoAge. Besides, Cox regression model indicated that both KDM-BA and PhenoAge were significantly associated with hazard of incident MASLD, with HRs being 1.047 (95% CI: 1.039-1.055) for KDM-BA and 1.047 (1.043-1.051) for PhenoAge (**Supplementary Table 5**). Mediation analyses demonstrated that both KDM-BA and PhenoAge significantly mediated the associations between all six TyG-related indices and MASLD incidence (**Figure 4** and **Supplementary Table 6**). For KDM-BA, mediated proportions were 3.68 (95% CI: 2.83, 4.68) for TyG-BMI, 2.90 (1.73, 3.96) for TyG-WC, 2.88 (2.01, 3.79) for TyG-WHtR, 3.73 (2.84, 4.80) for TyG-BRI, 3.23 (2.57, 3.89) for TyG-ABSI, and 3.12 (2.46, 3.81) for TyG-WWI, respectively (**Figure 4**). For PhenoAge, the corresponding proportions were 11.49 (95% CI: 9.64, 13.29), 9.36 (7.71, 10.62), 8.96 (7.42, 10.49), 12.03 (10.62, 13.60), 9.16 (8.26, 10.25), and 9.02 (7.83, 10.05), respectively (**Figure 4**).

### 3.5 Associations of novel TyG-related indices with prevalent PDFF-defined MASLD

**Figure 5:**
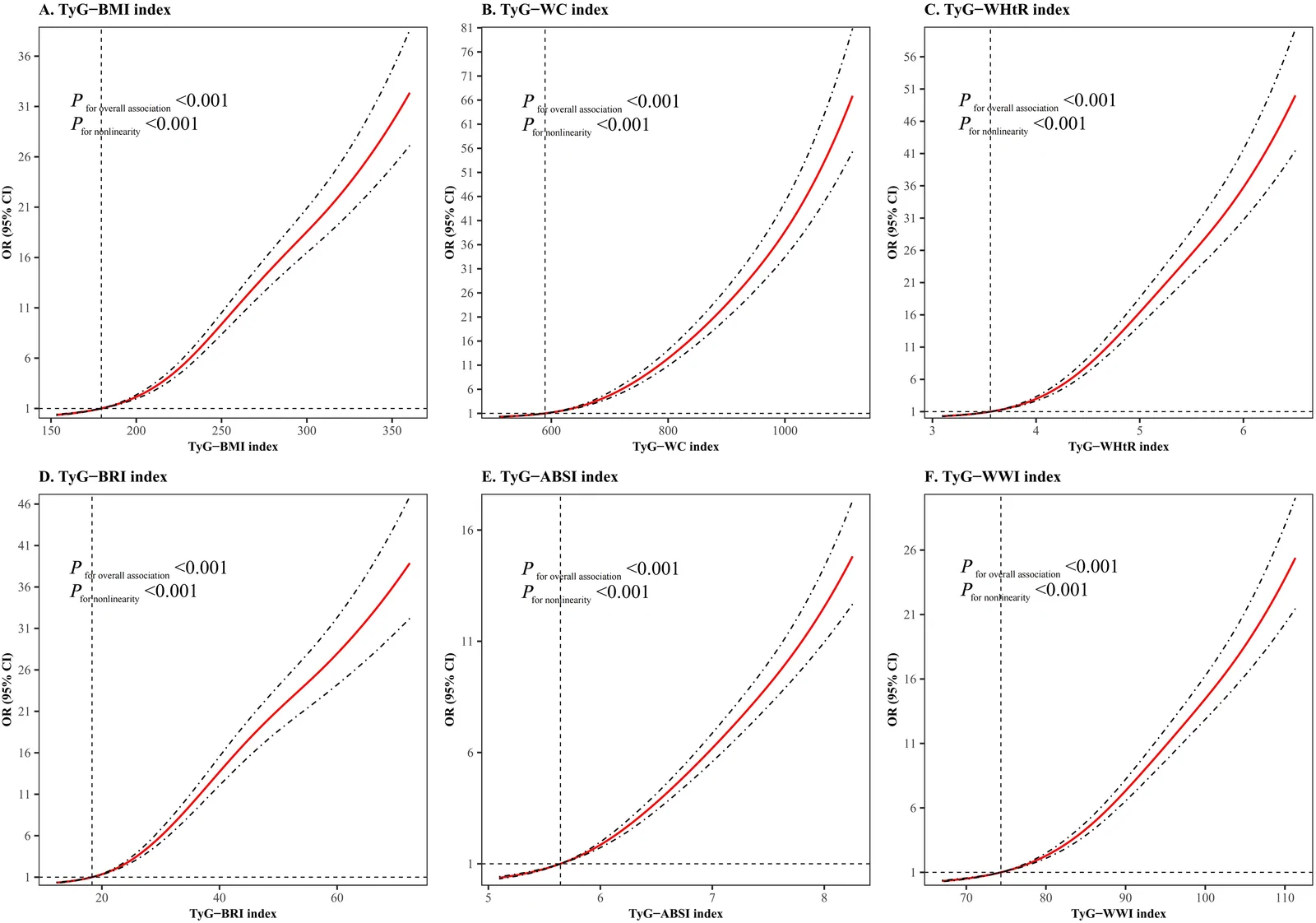
Restricted cubic spline analyses of six novel TyG and adiposity-related indices in relation to MASLD defined by PDFF ≥5% **(A)** TyG-BMI index, **(B)** TyG-WC index, **(C)** TyG-WHtR index, **(D)** TyG-BRI index, **(E)** TyG-ABSI index, **(F)** TyG-WWI index). Models were adjusted for age, sex, ethnicity, employed status, educational levels, Townsend deprivation index, family income, physical activity, smoking status, alcoholic drinking, sleeping duration, health diet score, total cholesterol, low density lipoprotein cholesterol, glycated hemoglobin, systolic/diastolic blood pressure, use of antihypertensive, antihyperglycemic, lipid-lowering medications, and self-reported history of hypertension, diabetes, and hyperlipidemia. OR odds ratio, CI confidence interval, MASLD metabolic dysfunction-associated steatotic liver disease; TyG triglycerides glucose index, BMI body mass index, WC waist circumference, WHtR waist-to-height ratio; BRI body roundness index; ABSI a body shape index; WWI weight-adjusted-waist index.

As presented in **Table 4**, elevated levels of six novel TyG-related indices were significantly associated with an increased odds of prevalent MASLD, defined as PDFF ≥5%. For per 1-SD increase in each index, the fully adjusted OR values (95% CI) for prevalent MASLD were 2.74 (2.65-2.85) for TyG-BMI, 3.12 (3.00-3.25) for TyG-WC, 3.04 (2.93-3.16) for TyG-WHtR, 2.79 (2.69-2.91) for TyG-BRI, 2.12 (2.05-2.20) for TyG-ABSI, and 2.52 (2.43-2.61) for TyG-WWI, respectively (**Table 4**). Additionally, compared with the lowest quartile group, ORs for the highest quartile group were 11.83 (10.74-13.04), 17.09 (15.34-19.06), 15.04 (13.59-16.67), 13.30 (12.03-14.71), 6.42 (5.84-7.07), and 9.41 (8.54-10.36), respectively (**Table 4**). RCS analyses further revealed non-linear associations between all six TyG-related indices and the risk of prevalent MASLD (**Figure 5**).

**Table 4:** Association between novel triglyceride glucose-related indices and risk of metabolic dysfunction-associated steatotic liver disease defined as PDFF≥5%.

Exposure	Cases/total population	Model 1		Model 2		Model 3	
		OR (95% CI)	*P* value	OR (95% CI)	*P* value	OR (95% CI)	*P* value
TyG-BMI index							
Per 1-SD increase		3.16 (3.06-3.27)	<0.001	3.07 (2.97-3.18)	<0.001	2.74 (2.65-2.85)	<0.001
Quartile 1	778/10,766	Reference		Reference		Reference	
Quartile 2	1,955/9,366	3.18 (2.91-3.48)	<0.001	3.09 (2.82-3.38)	<0.001	2.75 (2.51-3.01)	<0.001
Quartile 3	3,214/8,257	7.49 (6.87-8.17)	<0.001	7.09 (6.50-7.74)	<0.001	5.81 (5.31-6.37)	<0.001
Quartile 4	3,624/6,143	17.26 (15.79-18.90)	<0.001	15.97 (14.58-17.50)	<0.001	11.83 (10.74-13.04)	<0.001
* P* for trend		<0.001		<0.001		<0.001	
TyG-WC index							
Per 1-SD increase		3.59 (3.46-3.73)	<0.001	3.49 (3.36-3.62)	<0.001	3.12 (3.00-3.25)	<0.001
Quartile 1	690/10,564	Reference		Reference		Reference	
Quartile 2	1,996/9,258	4.56 (4.15-5.01)	<0.001	4.40 (4.01-4.84)	<0.001	3.84 (3.49-4.23)	<0.001
Quartile 3	3,183/8,306	11.26 (10.23-12.40)	<0.001	10.63 (9.65-11.71)	<0.001	8.44 (7.64-9.34)	<0.001
Quartile 4	3,702/6,404	25.91 (23.42-28.69)	<0.001	23.67 (21.37-26.25)	<0.001	17.09 (15.34-19.06)	<0.001
* P* for trend		<0.001		<0.001		<0.001	
TyG-WHtR index							
Per 1-SD increase		3.45 (3.33-3.57)	<0.001	3.35 (3.23-3.48)	<0.001	3.04 (2.93-3.16)	<0.001
Quartile 1	734/11,181	Reference		Reference		Reference	
Quartile 2	2,102/9,464	4.08 (3.72-4.47)	<0.001	3.94 (3.60-4.32)	<0.001	3.49 (3.18-3.83)	<0.001
Quartile 3	3,309/8,176	9.73 (8.90-10.66)	<0.001	9.21 (8.41-10.09)	<0.001	7.57 (6.89-8.32)	<0.001
Quartile 4	3,426/5,711	21.64 (19.69-23.81)	<0.001	19.94 (18.11-21.97)	<0.001	15.04 (13.59-16.67)	<0.001
* P* for trend		<0.001		<0.001		<0.001	
TyG-BRI index							
Per 1-SD increase		3.29 (3.17-3.41)	<0.001	3.18 (3.07-3.30)	<0.001	2.79 (2.69-2.91)	<0.001
Quartile 1	752/11,144	Reference		Reference		Reference	
Quartile 2	2,222/9,607	4.07 (3.73-4.46)	<0.001	3.94 (3.61-4.32)	<0.001	3.46 (3.16-3.80)	<0.001
Quartile 3	3,287/8,168	9.12 (8.34-9.98)	<0.001	8.60 (7.86-9.41)	<0.001	6.92 (6.31-7.60)	<0.001
Quartile 4	3,310/5,613	19.87 (18.10-21.84)	<0.001	18.24 (16.59-20.08)	<0.001	13.30 (12.03-14.71)	<0.001
* P* for trend		<0.001		<0.001		<0.001	
TyG-ABSI index							
Per 1-SD increase		2.41 (2.33-2.49)	<0.001	2.32 (2.25-2.40)	<0.001	2.12 (2.05-2.20)	<0.001
Quartile 1	1,088/10,455	Reference		Reference		Reference	
Quartile 2	2,208/8,983	2.98 (2.74-3.23)	<0.001	2.88 (2.65-3.12)	<0.001	2.47 (2.27-2.68)	<0.001
Quartile 3	2,952/8,310	5.20 (4.78-5.66)	<0.001	4.95 (4.55-5.39)	<0.001	3.91 (3.58-4.27)	<0.001
Quartile 4	3,323/6,784	9.32 (8.52-10.20)	<0.001	8.46 (7.73-9.27)	<0.001	6.42 (5.84-7.07)	<0.001
* P* for trend		<0.001		<0.001		<0.001	
TyG-WWI index							
Per 1-SD increase		2.85 (2.76-2.95)	<0.001	2.75 (2.66-2.85)	<0.001	2.52 (2.43-2.61)	<0.001
Quartile 1	925/11,119	Reference		Reference		Reference	
Quartile 2	2,223/9,307	3.54 (3.26-3.86)	<0.001	3.42 (3.14-3.72)	<0.001	2.97 (2.72-3.24)	<0.001
Quartile 3	3,241/8,219	7.45 (6.85-8.10)	<0.001	7.02 (6.45-7.65)	<0.001	5.59 (5.12-6.11)	<0.001
Quartile 4	3,182/5,887	13.68 (12.51-14.98)	<0.001	12.38 (11.30-13.57)	<0.001	9.41 (8.54-10.36)	<0.001
* P* for trend		<0.001		<0.001		<0.001	

Model 1 were adjusted for age and sex.

Model 2 were adjusted for age, sex, ethnicity, employed status, educational levels, Townsend deprivation index, family income, physical activity, smoking status, alcoholic drinking, sleeping duration, and health diet score.

Model 3 were adjusted for age, sex, ethnicity, employed status, educational levels, Townsend deprivation index, family income, physical activity, smoking status, alcoholic drinking, sleeping duration, health diet score, total cholesterol, low density lipoprotein cholesterol, glycated hemoglobin, systolic/diastolic blood pressure, use of antihypertensive, antihyperglycemic, lipid-lowering medications, and self-reported history of hypertension, diabetes, and hyperlipidemia.

PDFF proton density fat fraction; OR odd ratio; CI confidence interval; SD standard deviation; TyG triglycerides glucose index, BMI body mass index, WC waist circumference, WHtR waist-to-height ratio; BRI body roundness index; ABSI a body shape index; WWI weight-adjusted-waist index.

### 3.6 Additional analyses

Sensitivity analyses further confirmed the primary results after imputing missing covariate data using multiple imputation by chained equations (**Supplementary Table 7**), excluding participants who developed the MASLD during the first 2 years of follow-up (**Supplementary Table 8**), removing individuals with pre-existing other liver diseases at baseline (**Supplementary Table 9**), and using a restricted definition for MASLD outcome (**Supplementary Table 10**). In subgroup analyses, all six novel TyG-related indices were consistently associated with an elevated risk of MASLD across virous subgroups (**Supplementary Figure 1-S7**), with more pronounced associations in females, nonsmokers, drinkers, and individuals free of hypertension, diabetes, and hyperlipidemia (*P* values for interaction <0.05, except for TyG-ABSI with smoking status). As shown in **Supplementary Figure 1**, when stratified by sex, participants in the highest quartile of TyG-related indices exhibited a substantially higher risk of incident MASLD compared with those in the lowest quartile, with stronger associations observed in females than in males. Specifically, in females, the hazard ratios (HRs) were 7.35 (95% CI: 6.34–8.51) for TyG-BMI, 8.57 (7.47–9.82) for TyG-WC, 9.02 (7.77–10.47) for TyG-WHtR, 8.31 (7.19–9.62) for TyG-BRI, 3.84 (3.42–4.32) for ABSI, and 6.40 (5.59–7.32) for TyG-WWI, whereas the corresponding HRs in males were 4.61 (3.90–5.46), 5.46 (4.12–7.23), 5.96 (4.81–7.38), 5.90 (4.75–7.32), 3.20 (2.52–4.07), and 5.06 (4.06–6.30), respectively (**Supplementary Figure 1**).

## 4 Discussion

Based on a large prospective cohort study of 426,831 participants, this study comprehensively investigated associations between six novel TyG and obesity-related compsite indices, including TyG-BMI, TyG-WC, TyG-WHtR, TyG-BRI, TyG-ABSI, and TyG-WWI, and the risk of incident MASLD, as well as the potential role of biological aging. Our findings showed that higher levels of six TyG-related indices were significantly and nonlinearly associated with an elevated risk of MASLD. Among these indices, TyG-WHtR and TyG-WC demonstrated the superior predictive performance for MASLD risk than other indices. Mediation analyses indicated that biological aging accounted for 3%-12% of associations between TyG-related indices and MASLD risk. Moreover, all six TyG-related indices were significantly associated with higher odds of prevalent MASLD, defined as MRI-measured PDFF ≥5%. These results were robust and consistent across multiple sociodemographic and clinical subgroups, as well as in sensitive analyses. Together, our findings highlighted the importance of measuring and monitoring TyG-related indices, particularly TyG-WHtR and TyG-WC, in the early detection of MASLD patients, and revelated the mediating role of biological aging in the pathway from IR to MASLD.

Previous studies have established associations between TyG-related indices and risk of multiple cardiometabolic morbidities and mortality in both the general population and individuals with pre-existing chronic conditions (31, 33, 46, 49–52). For example, a recent meta-analysis of 85 studies summarized that five TyG and obesity-related composite indices, including TyG-BMI, TyG-WC, TyG-WHtR, TyG-BRI, and TyG-ABSI, were generally associated with elevated risk of overall CVD, coronary artery disease, stroke, and all-cause and cardiovascular mortality (52). For per 1-SD increase in these indices, harmonized relative risks (RRs) of incident total CVD were 1.17 (95% CI: 1.13-1.22) for TyG-BMI, 1.20 (1.16-1.25) for TyG-WC, 1.19 (1.15-1.23) for TyG-WHtR, and 1.12 (1.00-1.25) for TyG-BRI. However, evidences regarding their associations with MASLD risk were limited. In a study of 377,757 participants from the UK Biobank study, Hou et al. reported that higher TyG index levels were positively related with incident non-alcoholic fatty liver disease (NAFLD, the former term for MASLD) (15), with the fully-adjusted HR of 1.53 (95% CI: 1.45-1.62) per 1-SD increase. Notably, this study focused solely on TyG index alone, and did not examine the associations and predictive performances for TyG-related and obesity composite indices (15). Besides, a cohort study of 25,366 Chinese adults from the Dalian Health Management Cohort (DHMC) found that higher levels of TyG-BMI (HR of per 1-SD increase: 2.13, 95%: 2.07-2.19), TyG-WC (2.26, 2.18-2.34), and TyG-WHtR (2.16, 2.09-2.23) were associated with an increased risk of MASLD (23). However, this study was largely limited by its smaller sample size, relatively short follow-up period, lack of adjustment for several key confounders (such as physical activity and diet), and restriction to Chinese population (23). In the present study, using a large-scaled cohort study of over 420,000 participants with a 13-year follow-up period, we demonstrated that all six TyG and adiposity-related composite indices were significantly associated with risk of incident MASLD. Additionally, these associations remained consistent and robust when MASLD was objectively defined using MRI-measured PDFF. Overall, our findings highlighted the potential utility of TyG-related indices, particularly TyG-WHtR and TyG-WC, as simple and cost-effective measurements for the early detection of MASLD in both clinical practice and epidemiological research.

Furthermore, the combination of TyG index and adiposity indices has been shown to improve the predictive values for the incidence and prognosis of cardiometabolic diseases (18, 20, 33). Our study comprehensively assessed, for the first time, the predictive value of six novel TyG-related and obesity indices for MASLD incidence. Our results indicate that incorporating TyG-related and obesity composite indices, particularly for TyG-WHtR and TyG-WC, into traditional risk factors significantly enhances predictive performance for MASLD risk, which was consistent with previous studies. For instance, Song et al. reported that TyG-BMI (C index = 0.768, 95% CI = 0.762-0.774) demonstrated superior predictive ability for MASLD onset, followed by TyG-WC (0.758, 0.752-0.764) and TyG-WHtR (0.745, 0.739-0.751). He and colleagues found that TyG-WC and TyG-WHtR provided the greatest incremental predictive values for total CVD, CHD, stroke, and mortality in individuals with metabolic syndrome (MetS) (46). Similarly, another cohort study found that TyG-WC and TyG-WHtR had considerably enhanced performance in predicting risk of cardiometabolic multimorbidity (53). Notably, we observed a weak association with TyG-ABSI than others, which may be explained by the following reasons. First, TyG itself is highly correlated with visceral adiposity and the associated insulin-resistant state. When combining with ABSI, TyG may capture much of the pathogenic information related to visceral fat, leaving limited independent risk information for ABSI to add. Besides, MASLD risk appears to be more strongly driven by absolute adiposity rather than by body shape normalized for height and weight (54). ABSI was developed to quantify the excess risk attributable to abdominal size beyond that expected for a given BMI and height; thus, in a population where BMI and WC are the dominant drivers, the independent contribution of ABSI may be attenuated. Additionally, several studies have reported the prognostic values of TyG-related and adiposity composite indices in MASLD patients (55–57). A study of 97,331 MASLD patients showed significant improvements in C-index, NRI, and IDI for TyG-related indices in predicting risk of CVD and mortality, with greatest gains observed for TyG-WC and TyG-WHtR (55). Likewise, an analysis of 8,208 adults from the National Health and Nutrition Examination Survey (NHANES) showed that TyG-WC and TyG-WHtR outperformed the TyG index alone in predicting all-cause and cardiovascular mortality in MASLD population (56). Taken together, our findings undoubtedly extend the predictive utility of TyG-related indices to MASLD incidence and support their integration into early risk assessment and prevention strategies for MASLD.

Despite the precise mechanisms underlying relationship between TyG-related indices and MASLD incidence remain unclear, our mediation analyses provide new insights by identifying biological aging, quantified by KDM-BA and PhenoAge, as a key mediator. Biological aging represents a holistic measurement of physiological aging, and has been linked to TyG-related indices and MASLD risk in previous studies. For example, a cross-sectional study revealed a nonlinear and positive relationships between TyG index and biological aging (58). Each 1-unit increase was associated a 1.64- and 0.40-years increase in KDM-BA and PhenoAge. Moreover, Zhao et al. reported a prospective association between biological aging and risk of NAFLD in a bio-cohort study (27). And individuals with accelerated biological aging were reported to be more susceptible to liver steatosis and fibrosis, objectively measured using transient elastography measurements of controlled attenuation parameter and liver stiffness measurements (28). Aligning with these studies, our study found that all six TyG-related indices were positively associated with both KDM-BA and PhenoAge, while both biological aging metrics were independently associated with subsequent MASLD risk. Furthermore, our study, for the first time, demonstrated that biological aging contributes to the pathway from IR to MASLD incidence. These findings suggest that biological aging may serve as a clinically relevant intermediary linking IR-related metabolic dysregulation to the development of MASLD, highlighting that accelerated aging is not merely a correlate of metabolic dysfunction but may be one of the pathways through which metabolic dysregulation is translated into hepatic disease. Mechanistically, elevated TyG-related indices reflect a cluster of adverse metabolic states including hypertriglyceridemia, hyperglycemia, central adiposity, and IR (31, 58, 59). These abnormalities may induce oxidative stress, chronic low-grade inflammation (such as interleukin-6 and tumor necrosis factor-alpha), adipokines (such as adiponectin and leptin), and alterations in the gut microbiome, all of which are hallmarks of biological aging and are also implicated in MASLD pathogenesis (1, 8, 60, 61). Beyond, MASLD pathogenesis involves a complex interplay of organelle dysfunction and programmed cell death pathways. Mitochondrial dysfunction, characterized by impaired fatty acid β-oxidation and excessive reactive oxygen species production, promotes lipotoxic lipid accumulation and hepatocyte injury (62). Simultaneously, endoplasmic reticulum stress activates the unfolded protein response, which can further aggravate insulin resistance and steatosis (63). Dysregulation of autophagy, a critical process for degrading lipid droplets and damaged organelles, contributes to the persistence of steatosis and hepatocellular damage (64). More recently, ferroptosis—a form of iron-dependent programmed cell death driven by lipid peroxidation—has emerged as a key mechanism in the transition from simple steatosis to steatohepatitis. Single-cell analysis technologies have revealed that, in hepatoma cells, ferritin light chain and caspase-8 are closely associated with ferroptosis-related gene signatures, providing mechanistic insights into how metabolic stress may activate cell death pathways that accelerate MASLD progression (65). These interconnected mechanisms underscore the need for integrated biomarkers, such as the TyG-derived indices in our study, which may capture the upstream metabolic disturbances driving these pathological cascades.

This large prospective cohort study is, to our knowledge, the first to comprehensively investigate the associations of novel TyG and adiposity-related composite indices in relation to the risk of incident MASLD and their predictive performance. The key strengths of this study include the large sample size, extended follow-up duration, rigorous adjustment for a wide range of confounders, and the consistency of the findings across diverse subgroups and sensitivity analyses. Additionally, the results were further validated when MASLD was defined using objectively measured MRI-derived PDFF ≥5%. Nevertheless, several limitations should be considered when interpreting these results. First, the observational study design precludes causal inference. Second, despite controlling for multiple covariates, the observed associations may still be subject to unmeasured or residual confounding. Third, TyG-related indices and biological aging metrics were assessed only at baseline, therefore, longitudinal dynamitic changes in these indices and their relationship with MASLD risk could not be evaluated. Future studies should incorporate repeated measurements to explore how time-varying changes in TyG-related composite indices and biological aging influence MASLD development. Finally, as most UK Biobank participants are of European ancestry and relatively healthy volunteers, the generalizability of our findings to other populations may be limited.

## 5 Conclusion

In conclusion, our study demonstrated that six novel TyG-related indices were significantly associated with the risk of incident MASLD, independent of sociodemographic characteristics, lifestyle behaviors, and clinical factors. When incorporating into conventional risk models, TyG-WHtR and TyG-WC showed superior predictive performance for MASLD risk. These findings suggest that routine assessment and monitoring of TyG-related indices may serve as a simple and effective approach for the early detection of individuals at high risk of MASLD. Additionally, biological aging partially mediated the associations between TyG-related indices and MASLD risk, thereby offering novel mechanistic insights into the pathways linking IR to MASLD development. Future studies are warranted to validate these findings and further elucidate the longitudinal trajectories of TyG-related indices, biological aging, and the onset and progression of MASLD.

## Data Availability

The raw data supporting the conclusions of this article will be made available by the authors, without undue reservation.
