# Supplementary material for: Associations between novel triglyceride glucose and adiposity-related indices and risk of metabolic dysfunction-associated steatotic liver disease: the mediating role of biological aging and evidence from the UK Biobank
Source: Front Endocrinol (Lausanne). 2026 Aug 11;17:1915819. doi: 10.3389/fendo.2026.1915819 (PMC13503529; doi:10.3389/fendo.2026.1915819)
Supplement: Supplementary file 1 [file SupplementaryFile1.docx]

**Table S1** Variables used in the construction of biological aging and related data fields in UK Biobank

| **Field ID in UK Biobank** | **Description in UK Biobank** | **Labels in this study** |
| --- | --- | --- |
| **KDM-BA** |  |  |
| 3063 | Forced expiratory volume in 1-second (FEV_1_) | FEV_1_ |
| 4080 | Systolic blood pressure, automated reading | Systolic blood pressure |
| 30600 | Albumin | Albumin |
| 30610 | Alkaline phosphatase | Alkaline phosphatase |
| 30670 | Urea | Blood urea nitrogen |
| 30700 | Creatinine | Creatinine |
| 30710 | C-reactive protein | C-reactive protein |
| 30750 | Glycated haemoglobin (HbA1c) | Glycated haemoglobin |
| 30690 | Cholesterol | Total cholesterol |
| **PhenoAge** |  |  |
| 30600 | Albumin | Albumin |
| 30610 | Alkaline phosphatase | Alkaline phosphatase |
| 30700 | Creatinine | Creatinine |
| 30710 | C-reactive protein | C-reactive protein |
| 30740 | Glucose | Blood Glucose |
| 30270 | Mean sphered cell volume | Mean cell volume |
| 30070 | Red blood cell (erythrocyte) distribution width | Red cell distribution width |
| 30000 | White blood cell (leukocyte) count | White blood cell |
| 30180 | Lymphocyte percentage | Lymphocyte |

Abbreviations: KDM-BA, Klemera-Doubal method Biological Age, FEV1, forced expiratory volume in 1-second, HbA1c, glycated haemoglobin.

**Table S2** Baseline characteristics of included population stratified by quartile of TyG-BMI

| **Characteristics** | **TyG-BMI index** | | | | ***P* value** |
| --- | --- | --- | --- | --- | --- |
|  | **Quartile 1** | **Quartile 2** | **Quartile 3** | **Quartile 4** |  |
| **n** | 106,708 | 106,707 | 106,708 | 106,708 |  |
| **Age, year** | 54.89±8.27 | 56.88 ±8.07 | 57.35±7.96 | 57.08±7.83 | <0.001 |
| **Female** | 74307 (69.64) | 56549 (52.99) | 47791 (44.79) | 51286 (48.06) | <0.001 |
| **Whites** | 101056 (94.70) | 100764 (94.43) | 100214 (93.91) | 100552 (94.23) | <0.001 |
| **Employed status** |  |  |  |  | <0.001 |
| Employed | 97356 (91.24) | 99092 (92.86) | 98632 (92.43) | 94921 (88.95) |  |
| Unemployed | 8859 (8.30) | 7128 (6.68) | 7527 (7.05) | 11185 (10.48) |  |
| Missing | 493 (0.46) | 487 (0.46) | 549 (0.51) | 602 (0.56) |  |
| **Educational levels** |  |  |  |  | <0.001 |
| University or college | 43858 (41.10) | 36502 (34.21) | 31552 (29.57) | 26031 (24.39) |  |
| Others | 61910 (58.02) | 69088 (64.75) | 73768 (69.13) | 79086 (74.11) |  |
| Missing | 940 (0.88) | 1117 (1.05) | 1388 (1.30) | 1591 (1.49) |  |
| **Townsend deprivation index** | -2.27 (-3.73, 0.26) | -2.35 (-3.75, 0.09) | -2.21 (-3.66, 0.39) | -1.72 (-3.41, 1.25) | <0.001 |
| **Family income** |  |  |  |  | <0.001 |
| Low | 16992 (15.92) | 18938 (17.75) | 21300 (19.96) | 25543 (23.94) |  |
| Intermediate | 46369 (43.45) | 47916 (44.90) | 47552 (44.56) | 46029 (43.14) |  |
| High | 28496 (26.70) | 24697 (23.14) | 22408 (21.00) | 18499 (17.34) |  |
| Missing | 14851 (13.92) | 15156 (14.20) | 15448 (14.48) | 16637 (15.59) |  |
| **Physical activity** |  |  |  |  | <0.001 |
| Inadequate | 16354 (15.33) | 17009 (15.94) | 18649 (17.48) | 21230 (19.90) |  |
| Adequate | 78938 (73.98) | 76821 (71.99) | 72453 (67.90) | 63121 (59.15) |  |
| Missing | 11416 (10.70) | 12877 (12.07) | 15606 (14.62) | 22357 (20.95) |  |
| **Smoking status** |  |  |  |  | <0.001 |
| Never smoking | 64172 (60.14) | 59691 (55.94) | 55636 (52.14) | 52714 (49.40) |  |
| Ever smoking | 30584 (28.66) | 35577 (33.34) | 39261 (36.79) | 42153 (39.50) |  |
| Current smoking | 11566 (10.84) | 10956 (10.27) | 11267 (10.56) | 11131 (10.43) |  |
| Missing | 386 (0.36) | 483 (0.45) | 544 (0.51) | 710 (0.67) |  |
| **Alcoholic drinking** |  |  |  |  | <0.001 |
| Never | 7668 (7.19) | 7437 (6.97) | 8180 (7.67) | 10748 (10.07) |  |
| Less than 3 times/month | 48211 (45.18) | 49285 (46.19) | 50906 (47.71) | 57879 (54.24) |  |
| Three times/week or more | 50636 (47.45) | 49779 (46.65) | 47398 (44.42) | 37808 (35.43) |  |
| Missing | 193 (0.18) | 206 (0.19) | 224 (0.21) | 273 (0.26) |  |
| **Daily sleep duration** |  |  |  |  | <0.001 |
| <7 hours/day | 23601 (22.12) | 24493 (22.95) | 26320 (24.67) | 30152 (28.26) |  |
| 7-9 hours/day | 81254 (76.15) | 80075 (75.04) | 77663 (72.78) | 72431 (67.88) |  |
| >9 hours/day | 1239 (1.16) | 1483 (1.39) | 1953 (1.83) | 3030 (2.84) |  |
| Missing | 614 (0.58) | 656 (0.61) | 772 (0.72) | 1095 (1.03) |  |
| **Healthy diet score** | 3.53±1.29 | 3.37±1.30) | 3.23±1.32) | 3.11±1.33) | <0.001 |
| **TC, mmol/L** | 5.56±1.03) | 5.74±1.11) | 5.79±1.17) | 5.69±1.24) | <0.001 |
| **LDL-C, mmol/L** | 3.35±0.78) | 3.60±0.84) | 3.68±0.89) | 3.61±0.92) | <0.001 |
| **HbA1C, %** | 34.36±4.34) | 35.07±4.67) | 35.98±5.68) | 38.84±9.34) | <0.001 |
| **SBP, mmHg** | 131.17±18.54) | 137.43±18.28) | 140.59±17.96) | 142.59±17.69) | <0.001 |
| **DBP, mmHg** | 77.96±9.68) | 81.47±9.62) | 83.83±9.69) | 85.96±9.82) | <0.001 |
| **MASLD incidence** | 399 (0.37) | 780 (0.73) | 1535 (1.44) | 3594 (3.37) | <0.001 |
| **Use of medications** |  |  |  |  |  |
| Antihypertensive medication | 9697 (9.09) | 17261 (16.18) | 24529 (22.99) | 37373 (35.02) | <0.001 |
| Antihyperglycemic medication | 993 (0.93) | 1776 (1.66) | 3263 (3.06) | 9895 (9.27) | <0.001 |
| Lipid-lowering medication | 8328 (7.80) | 15037 (14.09) | 21094 (19.77) | 29854 (27.98) | <0.001 |
| **Self-reported disease history** |  |  |  |  |  |
| Hypertension | 14330 (13.43) | 23683 (22.19) | 32374 (30.34) | 46892 (43.94) | <0.001 |
| Diabetes | 1475 (1.38) | 2649 (2.48) | 4766 (4.47) | 13414 (12.57) | <0.001 |
| Hyperlipidemia | 6324 (5.93) | 11090 (10.39) | 15432 (14.46) | 19972 (18.72) | <0.001 |

Data were described as mean ± standard deviation (SD) for normally-distributed continue variables and median and interquartile ranges (IQR) for skewedly distributed continue variables, and categorical variables were described as number and precent (%). Missing covariate data were imputed using additional category “Missing” for categorical variables and median values for continue variables.

*Abbreviations*: TyG triglycerides-glucose index, BMI body mass index, TC total cholesterol; LDL-C low density lipoprotein cholesterol; HbA1C glycated hemoglobin, SBP systolic blood pressure; DBP diastolic blood pressure, MASLD metabolic dysfunction-associated steatotic liver disease.

**Table S3** Baseline characteristics of included population according to prevalent MASLD defined by PDFF ≥5%

| **Characteristics** | **Overall population** | **MASLD defined by PDFF ≥5%** | | ***P* value** |
| --- | --- | --- | --- | --- |
|  |  | Yes | No |  |
| **n** | 34,532 | 9,571 | 24,961 |  |
| **Age, year** | 55.04±7.52 | 55.21±7.33 | 54.98±7.60 | 0.009 |
| **Female** | 17,881 (51.78) | 3,785 (39.55) | 14,096 (56.47) | <0.001 |
| **Whites** | 33,411 (96.75) | 9,242 (96.56) | 24,169 (96.83) | 0.227 |
| **Employed status** |  |  |  | 0.383 |
| Employed | 32,689 (94.66) | 9,051 (94.57) | 23,638 (94.70) |  |
| Unemployed | 1,777 (5.15) | 506 (5.29) | 1,271 (5.09) |  |
| Missing | 66 (0.19) | 14 (0.15) | 52 (0.21) |  |
| **Educational levels** |  |  |  | <0.001 |
| University or college | 16,181 (46.86) | 3,829 (40.01) | 12,352 (49.49) |  |
| Others | 18,242 (52.83) | 5,710 (59.66) | 12,532 (50.21) |  |
| Missing | 109 (0.32) | 32 (0.33) | 77 (0.31) |  |
| **Townsend deprivation index** | -2.61 (-3.88, -0.48) | -2.52 (-3.83, -0.27) | -2.65 (-3.90, -0.57) | <0.001 |
| **Family income** |  |  |  | <0.001 |
| Low | 3,652 (10.58) | 1,102 (11.51) | 25,50 (10.22) |  |
| Intermediate | 1,6384 (47.45) | 4,630 (48.38) | 11,754 (47.09) |  |
| High | 11,598 (33.59) | 3,069 (32.07) | 8,529 (34.17) |  |
| Missing | 2,898 (8.39) | 770 (8.05) | 2,128 (8.53) |  |
| **Physical activity** |  |  |  | <0.001 |
| Inadequate | 5,315 (15.39) | 1,594 (16.65) | 3,721 (14.91) |  |
| Adequate | 25,372 (73.47) | 6,576 (68.71) | 18,796 (75.30) |  |
| Missing | 3,845 (11.13) | 1,401 (14.64) | 2,444 (9.79) |  |
| **Smoking status** |  |  |  | <0.001 |
| Never smoking | 20,977 (60.75) | 5,359 (55.99) | 15,618 (62.57) |  |
| Ever smoking | 11,362 (32.90) | 3,432 (35.86) | 7,930 (31.77) |  |
| Current smoking | 2,121 (6.14) | 756 (7.90) | 1,365 (5.47) |  |
| Missing | 72 (0.21) | 24 (0.25) | 48 (0.19) |  |
| **Alcoholic drinking** |  |  |  | <0.001 |
| Never | 1,606 (4.65) | 466 (4.87) | 1,140 (4.57) |  |
| Less than 3 times/month | 15,374 (44.52) | 4,318 (45.12) | 11,056 (44.29) |  |
| Three times/week or more | 17,536 (50.78) | 4,784 (49.98) | 12,752 (51.09) |  |
| Missing | 16 (0.05) | 3 (0.03) | 13 (0.05) |  |
| **Daily sleep duration** |  |  |  | <0.001 |
| <7 hours/day | 7,384 (21.38) | 2,324 (24.28) | 5,060 (20.27) |  |
| 7-9 hours/day | 26,746 (77.45) | 7,100 (74.18) | 19,646 (78.71) |  |
| >9 hours/day | 336 (0.97) | 130 (1.36) | 206 (0.83) |  |
| Missing | 66 (0.19) | 17 (0.18) | 49 (0.20) |  |
| **Healthy diet score** | 3.38±1.30 | 3.13±1.30 | 3.47±1.28 | <0.001 |
| **TyG-BMI index** | 224.39 (198.27, 255.03) | 254.95 (229.40, 283.87) | 213.56 (191.27, 240.29) | <0.001 |
| **TyG-WC index** | 762.32±137.25 | 856.37±125.05 | 726.26±123.99 | <0.001 |
| **TyG-WHtR index** | 4.49±0.75 | 5.01±0.69 | 4.29±0.67 | <0.001 |
| **TyG-BRI index** | 31.29 (23.60, 40.11) | 39.80 (32.71, 48.33) | 28.07 (21.62, 35.87) | <0.001 |
| **TyG-ABSI index** | 6.57±0.71 | 6.94±0.64 | 6.43±0.69 | <0.001 |
| **TyG-WWI index** | 87.02±9.84 | 93.04±8.67 | 84.71±9.26 | <0.001 |
| **TC, mmol/L** | 5.73±1.08 | 5.77±1.13 | 5.71±1.06 | <0.001 |
| **LDL-C, mmol/L** | 3.58±0.83 | 3.68±0.86 | 3.55±0.81 | <0.001 |
| **HbA1C, %** | 35.03±5.00 | 36.07±6.05 | 34.63±4.47 | <0.001 |
| **SBP, mmHg** | 135.11±17.65 | 139.41±17.06 | 133.46±17.60 | <0.001 |
| **DBP, mmHg** | 81.46±9.88 | 84.60±9.57 | 80.25±9.73 | <0.001 |
| **Use of medications** |  |  |  |  |
| Antihypertensive medication | 4,692 (13.59) | 1,979 (20.68) | 2,713 (10.87) | <0.001 |
| Antihyperglycemic medication | 630 (1.82) | 325 (3.40) | 305 (1.22) | <0.001 |
| Lipid-lowering medication | 4,097 (11.86) | 1,678 (17.53) | 2,419 (9.69) | <0.001 |
| **Self-reported disease history** |  |  |  |  |
| Hypertension | 6,852 (19.84) | 2,816 (29.42) | 4,036 (16.17) | <0.001 |
| Diabetes | 925 (2.68) | 499 (5.21) | 426 (1.71) | <0.001 |
| Hyperlipidemia | 3,088 (8.94) | 1,222 (12.77) | 1,866 (7.48) | <0.001 |

Data were described as mean ± standard deviation (SD) for normally-distributed continue variables and median and interquartile ranges (IQR) for skewedly distributed continue variables, and categorical variables were described as number and precent (%). Missing covariate data were imputed using additional category “Missing” for categorical variables and median values for continue variables.

*Abbreviations*: TyG triglycerides- glucose index, BMI body mass index, WC waist circumference, WHtR waist-to-height ratio; BRI body roundness index; ABSI a body shape index; WWI weight-adjusted-waist index, TC total cholesterol; LDL-C low density lipoprotein cholesterol; HbA1C glycated hemoglobin, SBP systolic blood pressure; DBP diastolic blood pressure.

| **Outcome/exposure** | **β (95% CI)** | | |
| --- | --- | --- | --- |
|  | **Model 1** | **Model 2** | **Model 3** |
| **KDM-BA** |  |  |  |
| TyG-BMI index | 0.92 (0.91-0.93) | 0.87 (0.86-0.88) | 0.51 (0.50-0.52) |
| TyG-WC index | 1.05 (1.04-1.06) | 0.99 (0.98-1.00) | 0.61 (0.60-0.62) |
| TyG-WHtR index | 0.98 (0.97-0.99) | 0.92 (0.91-0.93) | 0.54 (0.53-0.55) |
| TyG-BRI index | 0.91 (0.90-0.92) | 0.85 (0.84-0.86) | 0.52 (0.51-0.53) |
| TyG-ABSI index | 0.78 (0.77-0.80) | 0.70 (0.69-0.71) | 0.30 (0.29-0.31) |
| TyG-WWI index | 0.89 (0.88-0.90) | 0.81 (0.80-0.82) | 0.40 (0.39-0.41) |
| **PhenoAge** |  |  |  |
| TyG-BMI index | 1.95 (1.94-1.97) | 1.76 (1.74-1.78) | 1.52 (1.51-1.54) |
| TyG-WC index | 2.14 (2.12-2.16) | 1.89 (1.88-1.91) | 1.62 (1.60-1.63) |
| TyG-WHtR index | 2.06 (2.04-2.07) | 1.82 (1.80-1.83) | 1.57 (1.56-1.59) |
| TyG-BRI index | 2.03 (2.02-2.05) | 1.80 (1.79-1.82) | 1.52 (1.50-1.54) |
| TyG-ABSI index | 1.50 (1.48-1.52) | 1.19 (1.17-1.21) | 1.07 (1.05-1.09) |
| TyG-WWI index | 1.78 (1.76-1.79) | 1.47 (1.46-1.49) | 1.19 (1.17-1.21) |

**Table S4** Associations between TyG-related indices and biological aging metrics

Data were presented as β with 95% CI per 1-SD increase in TyG-related indices, estimated from multivariate adjusted linear regression models. Model 1 were adjusted for age and sex. Model 2 were adjusted for age, sex, ethnicity, employed status, educational levels, Townsend deprivation index, family income, physical activity, smoking status, alcoholic drinking, sleeping duration, and health diet score. Model 3 were adjusted for age, sex, ethnicity, employed status, educational levels, Townsend deprivation index, family income, physical activity, smoking status, alcoholic drinking, sleeping duration, health diet score, total cholesterol, low density lipoprotein cholesterol, glycated hemoglobin, systolic/diastolic blood pressure, use of antihypertensive, antihyperglycemic, lipid-lowering medications, and self-reported history of hypertension, diabetes, and hyperlipidemia.

*Abbreviations*: CI confidence interval, SD standard deviation; KDM-BA Klemera-Doubal method Biological Age; TyG triglycerides glucose index, BMI body mass index, WC waist circumference, WHtR waist-to-height ratio; BRI body roundness index; ABSI a body shape index; WWI weight-adjusted-waist index.

**Table S5** Association between biological aging metrics and risk of metabolic dysfunction-associated steatotic liver disease

| **Biological aging** | **HR (95% CI)** | | |
| --- | --- | --- | --- |
|  | **Model 1** | **Model 2** | **Model 3** |
| KDM-BA | 1.090 (1.083-1.097) | 1.068 (1.061-1.075) | 1.047 (1.039-1.055) |
| PhenoAge | 1.076 (1.073-1.078) | 1.062 (1.058-1.065) | 1.047 (1.043-1.051) |

Data were presented as HR with 95% CI per 1-unite increase in biological aging metrics, estimated from multivariate adjusted Cox regression models. Model 1 were adjusted for age and sex. Model 2 were adjusted for age, sex, ethnicity, employed status, educational levels, Townsend deprivation index, family income, physical activity, smoking status, alcoholic drinking, sleeping duration, and health diet score. Model 3 were adjusted for age, sex, ethnicity, employed status, educational levels, Townsend deprivation index, family income, physical activity, smoking status, alcoholic drinking, sleeping duration, health diet score, total cholesterol, low density lipoprotein cholesterol, glycated hemoglobin, systolic/diastolic blood pressure, use of antihypertensive, antihyperglycemic, lipid-lowering medications, and self-reported history of hypertension, diabetes, and hyperlipidemia.

*Abbreviations*: HR hazard ratio; CI confidence interval, KDM-BA Klemera-Doubal method Biological Age.

**Table S6** Mediated role of biological aging in the association between TyG-related indices and MASLD risk

| **Exposure/mediators** | **Total effect** | **Direct effect** | **Indirect effect** | **Mediated proportion (%)** |
| --- | --- | --- | --- | --- |
| TyG-BMI index |  |  |  |  |
| KDM-DA | 1.5888 (1.5611, 1.6261) | 1.5671 (1.5380, 1.6054) | 1.0138 (1.0106, 1.0174) | 3.68 (2.83, 4.68) |
| PhenoAge | 1.5869 (1.5586, 1.6206) | 1.5195 (1.4900, 1.5583) | 1.0444 (1.0374, 1.0503) | 11.49 (9.64, 13.29) |
| TyG-WC index |  |  |  |  |
| KDM-DA | 1.8355 (1.7998, 1.8858) | 1.8113 (1.7726, 1.8628) | 1.0134 (1.0082, 1.0187) | 2.90 (1.73, 3.96) |
| PhenoAge | 1.8307 (1.7839, 1.8835) | 1.7530 (1.7074, 1.8086) | 1.0443 (1.0362, 1.0501) | 9.36 (7.71, 10.62) |
| TyG-WHtR index |  |  |  |  |
| KDM-DA | 1.7923 (1.7575, 1.8274) | 1.7696 (1.7332, 1.8040) | 1.0129 (1.0091, 1.0171) | 2.88 (2.01, 3.79) |
| PhenoAge | 1.7870 (1.7544, 1.8236) | 1.7165 (1.6749, 1.7525) | 1.0411 (1.0336, 1.0482) | 8.96 (7.42, 10.49) |
| TyG-BRI index |  |  |  |  |
| KDM-DA | 1.5584 (1.5322, 1.5834) | 1.5375 (1.5121, 1.5630) | 1.0136 (1.0103, 1.0173) | 3.73 (2.84, 4.80) |
| PhenoAge | 1.5639 (1.5365, 1.5944) | 1.4961 (1.4739, 1.5291) | 1.0454 (1.0402, 1.0516) | 12.03 (10.62, 13.60) |
| TyG-ABSI index |  |  |  |  |
| KDM-DA | 1.5922 (1.5466, 1.6332) | 1.5731 (1.5290, 1.6138) | 1.0122 (1.0098, 1.0145) | 3.23 (2.57, 3.89) |
| PhenoAge | 1.5755 (1.5295, 1.6190) | 1.5228 (1.4750, 1.5644) | 1.0346 (1.0315, 1.0385) | 9.16 (8.26, 10.25) |
| TyG-WWI index |  |  |  |  |
| KDM-DA | 1.7537 (1.7058, 1.7987) | 1.7302 (1.6823, 1.7730) | 1.0136 (1.0107, 1.0167) | 3.12 (2.46, 3.81) |
| PhenoAge | 1.7402 (1.6960, 1.7824) | 1.6734 (1.6293, 1.7168) | 1.0399 (1.0347, 1.0440) | 9.02 (7.83, 10.05) |

Models were adjusted for age, sex, ethnicity, employed status, educational levels, Townsend deprivation index, family income, physical activity, smoking status, alcoholic drinking, sleeping duration, health diet score, total cholesterol, low density lipoprotein cholesterol, glycated hemoglobin, systolic/diastolic blood pressure, use of antihypertensive, antihyperglycemic, lipid-lowering medications, and self-reported history of hypertension, diabetes, and hyperlipidemia.

*Abbreviations*: KDM-BA Klemera-Doubal method Biological Age; TyG triglycerides glucose index, BMI body mass index, WC waist circumference, WHtR waist-to-height ratio; BRI body roundness index; ABSI a body shape index; WWI weight-adjusted-waist index.

**Table S7** Sensitivity analysis for associations between novel triglyceride glucose-related indices and risk of MASLD: imputing missing covariate data using multiple imputation

| **Exposure** | **Cases/incidence rate** | **HR (95% CI)** | | |
| --- | --- | --- | --- | --- |
|  |  | **Model 1** | **Model2** | **Model 3** |
| TyG-BMI index |  |  |  |  |
| Per 1-SD increase | - | 1.86 (1.83-1.89) | 1.73 (1.70-1.77) | 1.59 (1.55-1.62) |
| Quartile 1 | 399/0.28 | Reference | Reference | Reference |
| Quartile 2 | 780/0.55 | 1.97 (1.75-2.23) | 1.91 (1.69-2.16) | 1.88 (1.66-2.12) |
| Quartile 3 | 1535/1.08 | 3.93 (3.52-4.39) | 3.60 (3.22-4.03) | 3.38 (3.01-3.78) |
| Quartile 4 | 3594/2.57 | 9.41 (8.48-10.44) | 7.80 (7.02-8.66) | 6.32 (5.66-7.07) |
| *P* for trend | - | <0.001 | <0.001 | <0.001 |
| TyG-WC index |  |  |  |  |
| Per 1-SD increase | - | 2.18 (2.13-2.23) | 2.01 (1.96-2.05) | 1.84 (1.79-1.89) |
| Quartile 1 | 370/0.26 | Reference | Reference | Reference |
| Quartile 2 | 890/0.62 | 2.77 (2.45-3.13) | 2.57 (2.27-2.90) | 2.51 (2.22-2.84) |
| Quartile 3 | 1613/1.14 | 5.65 (5.04-6.34) | 4.94 (4.40-5.54) | 4.58 (4.07-5.16) |
| Quartile 4 | 3435/2.47 | 13.22 (11.83-14.78) | 10.41 (9.31-11.65) | 8.33 (7.40-9.38) |
| *P* for trend | - | <0.001 | <0.001 | <0.001 |
| TyG-WHtR index |  |  |  |  |
| Per 1-SD increase | - | 2.10 (2.06-2.14) | 1.94 (1.90-1.98) | 1.79 (1.75-1.84) |
| Quartile 1 | 334/0.23 | Reference | Reference | Reference |
| Quartile 2 | 779/0.54 | 2.50 (2.19-2.84) | 2.36 (2.07-2.69) | 2.35 (2.06-2.67) |
| Quartile 3 | 1579/1.11 | 5.27 (4.67-5.93) | 4.69 (4.16-5.28) | 4.48 (3.96-5.07) |
| Quartile 4 | 3616/2.6 | 12.44 (11.10-13.94) | 9.94 (8.86-11.15) | 8.24 (7.30-9.31) |
| *P* for trend | - | <0.001 | <0.001 | <0.001 |
| TyG-BRI index |  |  |  |  |
| Per 1-SD increase | - | 1.81 (1.78-1.83) | 1.70 (1.67-1.73) | 1.56 (1.53-1.59) |
| Quartile 1 | 339/0.23 | Reference | Reference | Reference |
| Quartile 2 | 765/0.53 | 2.39 (2.11-2.72) | 2.28 (2.00-2.59) | 2.24 (1.97-2.55) |
| Quartile 3 | 1593/1.12 | 5.18 (4.60-5.83) | 4.64 (4.12-5.23) | 4.35 (3.85-4.92) |
| Quartile 4 | 3611/2.6 | 12.08 (10.79-13.53) | 9.69 (8.64-10.86) | 7.85 (6.97-8.85) |
| *P* for trend | - | <0.001 | <0.001 | <0.001 |
| TyG-ABSI index |  |  |  |  |
| Per 1-SD increase | - | 1.92 (1.87-1.97) | 1.74 (1.70-1.79) | 1.59 (1.55-1.64) |
| Quartile 1 | 632/0.44 | Reference | Reference | Reference |
| Quartile 2 | 1140/0.8 | 2.04 (1.85-2.25) | 1.86 (1.69-2.06) | 1.75 (1.58-1.93) |
| Quartile 3 | 1708/1.21 | 3.41 (3.10-3.75) | 2.94 (2.68-3.24) | 2.59 (2.35-2.86) |
| Quartile 4 | 2828/2.04 | 6.29 (5.73-6.90) | 4.94 (4.50-5.43) | 3.89 (3.52-4.29) |
| *P* for trend | - | <0.001 | <0.001 | <0.001 |
| TyG-WWI index |  |  |  |  |
| Per 1-SD increase | - | 2.08 (2.03-2.13) | 1.89 (1.85-1.94) | 1.76 (1.71-1.81) |
| Quartile 1 | 414/0.29 | Reference | Reference | Reference |
| Quartile 2 | 979/0.68 | 2.60 (2.31-2.91) | 2.39 (2.13-2.69) | 2.30 (2.05-2.59) |
| Quartile 3 | 1632/1.15 | 4.59 (4.11-5.12) | 3.98 (3.56-4.44) | 3.65 (3.26-4.08) |
| Quartile 4 | 3283/2.36 | 9.74 (8.77-10.83) | 7.57 (6.80-8.42) | 6.11 (5.47-6.84) |
| *P* for trend | - | <0.001 | <0.001 | <0.001 |

Model 1 were adjusted for age and sex.

Model 2 were adjusted for age, sex, ethnicity, employed status, educational levels, Townsend deprivation index, family income, physical activity, smoking status, alcoholic drinking, sleeping duration, and health diet score.

Model 3 were adjusted for age, sex, ethnicity, employed status, educational levels, Townsend deprivation index, family income, physical activity, smoking status, alcoholic drinking, sleeping duration, health diet score, total cholesterol, low density lipoprotein cholesterol, glycated hemoglobin, systolic/diastolic blood pressure, use of antihypertensive, antihyperglycemic, lipid-lowering medications, and self-reported history of hypertension, diabetes, and hyperlipidemia.

*Abbreviations*: MASLD metabolic dysfunction-associated steatotic liver disease; HR hazard ratio, CI confidence interval, SD standard deviation; TyG triglycerides- glucose index, BMI body mass index, WC waist circumference, WHtR waist-to-height ratio; BRI body roundness index; ABSI a body shape index; WWI weight-adjusted-waist index.

**Table S8** Sensitivity analysis for associations between novel triglyceride glucose-related indices and risk of MASLD: excluding individuals who developed MASLD within the first 2 years of follow up (n=263)

| **Exposure** | **Cases/incidence rate** | **HR (95% CI)** | | |
| --- | --- | --- | --- | --- |
|  |  | **Model 1** | **Model2** | **Model 3** |
| TyG-BMI index |  |  |  |  |
| Per 1-SD increase | - | 1.85 (1.82-1.88) | 1.72 (1.69-1.76) | 1.58 (1.55-1.62) |
| Quartile 1 | 383/0.27 | Reference | Reference | Reference |
| Quartile 2 | 751/0.53 | 1.98 (1.75-2.24) | 1.92 (1.69-2.17) | 1.89 (1.67-2.14) |
| Quartile 3 | 1489/1.05 | 3.98 (3.55-4.45) | 3.63 (3.24-4.07) | 3.43 (3.06-3.86) |
| Quartile 4 | 3422/2.44 | 9.35 (8.41-10.40) | 7.67 (6.89-8.55) | 6.32 (5.65-7.08) |
| *P* for trend |  | <0.001 | <0.001 | <0.001 |
| TyG-WC index |  |  |  |  |
| Per 1-SD increase | - | 2.17 (2.12-2.22) | 1.99 (1.95-2.04) | 1.83 (1.78-1.88) |
| Quartile 1 | 358/0.25 | Reference | Reference | Reference |
| Quartile 2 | 856/0.6 | 2.76 (2.44-3.12) | 2.55 (2.25-2.89) | 2.51 (2.21-2.84) |
| Quartile 3 | 1559/1.1 | 5.65 (5.03-6.35) | 4.92 (4.37-5.53) | 4.61 (4.08-5.20) |
| Quartile 4 | 3272/2.35 | 13.05 (11.65-14.61) | 10.16 (9.07-11.40) | 8.27 (7.33-9.33) |
| *P* for trend |  | <0.001 | <0.001 | <0.001 |
| TyG-WHtR index |  |  |  |  |
| Per 1-SD increase | - | 2.09 (2.05-2.13) | 1.93 (1.88-1.97) | 1.79 (1.74-1.83) |
| Quartile 1 | 323/0.22 | Reference | Reference | Reference |
| Quartile 2 | 752/0.52 | 2.49 (2.19-2.84) | 2.35 (2.06-2.68) | 2.35 (2.06-2.68) |
| Quartile 3 | 1527/1.08 | 5.27 (4.67-5.95) | 4.67 (4.13-5.28) | 4.51 (3.98-5.11) |
| Quartile 4 | 3443/2.47 | 12.28 (10.94-13.79) | 9.71 (8.63-10.92) | 8.18 (7.23-9.26) |
| *P* for trend |  | <0.001 | <0.001 | <0.001 |
| TyG-BRI index |  |  |  |  |
| Per 1-SD increase | - | 1.80 (1.77-1.83) | 1.69 (1.66-1.72) | 1.55 (1.52-1.58) |
| Quartile 1 | 328/0.23 | Reference | Reference | Reference |
| Quartile 2 | 738/0.51 | 2.39 (2.10-2.72) | 2.27 (1.99-2.59) | 2.24 (1.97-2.56) |
| Quartile 3 | 1540/1.08 | 5.18 (4.59-5.84) | 4.62 (4.09-5.22) | 4.37 (3.86-4.95) |
| Quartile 4 | 3439/2.47 | 11.92 (10.63-13.38) | 9.46 (8.42-10.64) | 7.79 (6.89-8.80) |
| *P* for trend |  | <0.001 | <0.001 | <0.001 |
| TyG-ABSI index |  |  |  |  |
| Per 1-SD increase | - | 1.91 (1.86-1.96) | 1.73 (1.68-1.77) | 1.59 (1.54-1.64) |
| Quartile 1 | 609/0.42 | Reference | Reference | Reference |
| Quartile 2 | 1098/0.77 | 2.04 (1.84-2.25) | 1.86 (1.68-2.05) | 1.76 (1.59-1.94) |
| Quartile 3 | 1639/1.16 | 3.40 (3.09-3.75) | 2.91 (2.65-3.21) | 2.60 (2.35-2.87) |
| Quartile 4 | 2699/1.94 | 6.24 (5.68-6.86) | 4.84 (4.40-5.33) | 3.87 (3.50-4.29) |
| *P* for trend |  | <0.001 | <0.001 | <0.001 |
| TyG-WWI index |  |  |  |  |
| Per 1-SD increase | - | 2.07 (2.02-2.12) | 1.88 (1.83-1.92) | 1.75 (1.70-1.80) |
| Quartile 1 | 406/0.28 | Reference | Reference | Reference |
| Quartile 2 | 940/0.66 | 2.54 (2.26-2.86) | 2.34 (2.08-2.63) | 2.26 (2.01-2.54) |
| Quartile 3 | 1572/1.11 | 4.51 (4.03-5.04) | 3.88 (3.47-4.34) | 3.60 (3.21-4.03) |
| Quartile 4 | 3127/2.25 | 9.48 (8.52-10.55) | 7.27 (6.52-8.10) | 5.96 (5.32-6.68) |
| *P* for trend |  | <0.001 | <0.001 | <0.001 |

Model 1 were adjusted for age and sex.

Model 2 were adjusted for age, sex, ethnicity, employed status, educational levels, Townsend deprivation index, family income, physical activity, smoking status, alcoholic drinking, sleeping duration, and health diet score.

Model 3 were adjusted for age, sex, ethnicity, employed status, educational levels, Townsend deprivation index, family income, physical activity, smoking status, alcoholic drinking, sleeping duration, health diet score, total cholesterol, low density lipoprotein cholesterol, glycated hemoglobin, systolic/diastolic blood pressure, use of antihypertensive, antihyperglycemic, lipid-lowering medications, and self-reported history of hypertension, diabetes, and hyperlipidemia.

*Abbreviations*: MASLD metabolic dysfunction-associated steatotic liver disease; HR hazard ratio, CI confidence interval, SD standard deviation; TyG triglycerides- glucose index, BMI body mass index, WC waist circumference, WHtR waist-to-height ratio; BRI body roundness index; ABSI a body shape index; WWI weight-adjusted-waist index.

**Table S9** Sensitivity analysis for associations between novel triglyceride glucose-related indices and risk of MASLD: excluding individuals with a prior diagnosis of other liver disease at baseline (n=5,902)

| **Exposure** | **Cases/incidence rate** | **HR (95% CI)** | | |
| --- | --- | --- | --- | --- |
|  |  | **Model 1** | **Model2** | **Model 3** |
| TyG-BMI index |  |  |  |  |
| Per 1-SD increase | - | 1.87 (1.84-1.90) | 1.74 (1.71-1.77) | 1.60 (1.57-1.63) |
| Quartile 1 | 355/0.25 | Reference | Reference | Reference |
| Quartile 2 | 744/0.53 | 2.13 (1.87-2.41) | 2.04 (1.80-2.32) | 2.00 (1.76-2.27) |
| Quartile 3 | 1452/1.03 | 4.20 (3.74-4.72) | 3.82 (3.39-4.29) | 3.56 (3.16-4.02) |
| Quartile 4 | 3455/2.5 | 10.22 (9.15-11.41) | 8.34 (7.46-9.32) | 6.78 (6.03-7.62) |
| *P* for trend |  | <0.001 | <0.001 | <0.001 |
| TyG-WC index |  |  |  |  |
| Per 1-SD increase | - | 2.19 (2.15-2.24) | 2.02 (1.97-2.06) | 1.86 (1.81-1.91) |
| Quartile 1 | 345/0.24 | Reference | Reference | Reference |
| Quartile 2 | 845/0.6 | 2.83 (2.50-3.21) | 2.62 (2.31-2.97) | 2.54 (2.24-2.89) |
| Quartile 3 | 1540/1.1 | 5.83 (5.18-6.57) | 5.07 (4.50-5.71) | 4.67 (4.13-5.27) |
| Quartile 4 | 3276/2.38 | 13.69 (12.20-15.35) | 10.66 (9.49-11.97) | 8.51 (7.53-9.62) |
| *P* for trend |  | <0.001 | <0.001 | <0.001 |
| TyG-WHtR index |  |  |  |  |
| Per 1-SD increase | - | 2.11 (2.07-2.15) | 1.95 (1.91-1.99) | 1.81 (1.76-1.85) |
| Quartile 1 | 312/0.22 | Reference | Reference | Reference |
| Quartile 2 | 723/0.51 | 2.49 (2.18-2.84) | 2.35 (2.05-2.68) | 2.31 (2.02-2.65) |
| Quartile 3 | 1526/1.09 | 5.48 (4.84-6.20) | 4.85 (4.28-5.48) | 4.59 (4.05-5.22) |
| Quartile 4 | 3445/2.5 | 12.74 (11.33-14.33) | 10.07 (8.93-11.34) | 8.33 (7.35-9.44) |
| *P* for trend |  | <0.001 | <0.001 | <0.001 |
| TyG-BRI index |  |  |  |  |
| Per 1-SD increase | - | 1.81 (1.78-1.84) | 1.70 (1.67-1.73) | 1.56 (1.53-1.59) |
| Quartile 1 | 318/0.22 | Reference | Reference | Reference |
| Quartile 2 | 710/0.5 | 2.38 (2.08-2.72) | 2.26 (1.97-2.58) | 2.20 (1.93-2.52) |
| Quartile 3 | 1525/1.09 | 5.31 (4.70-6.01) | 4.74 (4.19-5.36) | 4.41 (3.89-5.00) |
| Quartile 4 | 3453/2.51 | 12.36 (11.00-13.89) | 9.80 (8.71-11.03) | 7.93 (7.01-8.97) |
| *P* for trend |  | <0.001 | <0.001 | <0.001 |
| TyG-ABSI index |  |  |  |  |
| Per 1-SD increase | - | 1.92 (1.87-1.97) | 1.73 (1.69-1.78) | 1.59 (1.55-1.64) |
| Quartile 1 | 606/0.42 | Reference | Reference | Reference |
| Quartile 2 | 1096/0.78 | 2.04 (1.85-2.26) | 1.87 (1.69-2.07) | 1.75 (1.58-1.93) |
| Quartile 3 | 1626/1.16 | 3.40 (3.09-3.74) | 2.92 (2.65-3.22) | 2.57 (2.32-2.83) |
| Quartile 4 | 2678/1.95 | 6.25 (5.68-6.87) | 4.87 (4.42-5.36) | 3.85 (3.48-4.26) |
| *P* for trend |  | <0.001 | <0.001 | <0.001 |
| TyG-WWI index |  |  |  |  |
| Per 1-SD increase | - | 2.08 (2.04-2.13) | 1.89 (1.85-1.94) | 1.76 (1.71-1.81) |
| Quartile 1 | 393/0.27 | Reference | Reference | Reference |
| Quartile 2 | 927/0.66 | 2.59 (2.30-2.92) | 2.39 (2.12-2.69) | 2.28 (2.03-2.57) |
| Quartile 3 | 1558/1.11 | 4.63 (4.14-5.18) | 4.00 (3.57-4.48) | 3.65 (3.25-4.09) |
| Quartile 4 | 3128/2.28 | 9.81 (8.81-10.93) | 7.56 (6.77-8.43) | 6.11 (5.45-6.86) |
| *P* for trend |  | <0.001 | <0.001 | <0.001 |

Model 1 were adjusted for age and sex.

Model 2 were adjusted for age, sex, ethnicity, employed status, educational levels, Townsend deprivation index, family income, physical activity, smoking status, alcoholic drinking, sleeping duration, and health diet score.

Model 3 were adjusted for age, sex, ethnicity, employed status, educational levels, Townsend deprivation index, family income, physical activity, smoking status, alcoholic drinking, sleeping duration, health diet score, total cholesterol, low density lipoprotein cholesterol, glycated hemoglobin, systolic/diastolic blood pressure, use of antihypertensive, antihyperglycemic, lipid-lowering medications, and self-reported history of hypertension, diabetes, and hyperlipidemia.

*Abbreviations*: HR hazard ratio, CI confidence interval, SD standard deviation; TyG triglycerides- glucose index, BMI body mass index, WC waist circumference, WHtR waist-to-height ratio; BRI body roundness index; ABSI a body shape index; WWI weight-adjusted-waist index.

**Table S10** Sensitivity analysis for associations between novel triglyceride glucose-related indices and risk of MASLD: using a restricted outcome definition

| **Exposure** | **HR (95% CI)** | | |
| --- | --- | --- | --- |
|  | **Model 1** | **Model2** | **Model 3** |
| TyG-BMI index |  |  |  |
| Per 1-SD increase | 1.84 (1.81-1.88) | 1.71 (1.68-1.75) | 1.58 (1.54-1.61) |
| Quartile 1 | Reference | Reference | Reference |
| Quartile 2 | 2.01 (1.77-2.28) | 1.94 (1.71-2.20) | 1.90 (1.67-2.15) |
| Quartile 3 | 3.99 (3.56-4.48) | 3.64 (3.25-4.09) | 3.41 (3.03-3.84) |
| Quartile 4 | 9.35 (8.39-10.41) | 7.67 (6.87-8.55) | 6.30 (5.62-7.06) |
| *P* for trend | <0.001 | <0.001 | <0.001 |
| TyG-WC index |  |  |  |
| Per 1-SD increase | 2.15 (2.10-2.20) | 1.97 (1.93-2.02) | 1.82 (1.78-1.87) |
| Quartile 1 | Reference | Reference | Reference |
| Quartile 2 | 2.80 (2.47-3.18) | 2.59 (2.28-2.93) | 2.52 (2.22-2.86) |
| Quartile 3 | 5.72 (5.08-6.44) | 4.97 (4.41-5.60) | 4.61 (4.08-5.21) |
| Quartile 4 | 13.05 (11.64-14.63) | 10.15 (9.04-11.40) | 8.23 (7.28-9.30) |
| *P* for trend | <0.001 | <0.001 | <0.001 |
| TyG-WHtR index |  |  |  |
| Per 1-SD increase | 2.08 (2.03-2.12) | 1.91 (1.87-1.95) | 1.78 (1.73-1.83) |
| Quartile 1 | Reference | Reference | Reference |
| Quartile 2 | 2.57 (2.25-2.93) | 2.42 (2.12-2.76) | 2.39 (2.09-2.74) |
| Quartile 3 | 5.42 (4.79-6.13) | 4.79 (4.23-5.42) | 4.57 (4.02-5.19) |
| Quartile 4 | 12.41 (11.03-13.96) | 9.79 (8.69-11.04) | 8.20 (7.23-9.30) |
| *P* for trend | <0.001 | <0.001 | <0.001 |
| TyG-BRI index |  |  |  |
| Per 1-SD increase | 1.79 (1.76-1.82) | 1.68 (1.65-1.71) | 1.55 (1.51-1.58) |
| Quartile 1 | Reference | Reference | Reference |
| Quartile 2 | 2.45 (2.14-2.79) | 2.32 (2.03-2.65) | 2.28 (1.99-2.60) |
| Quartile 3 | 5.31 (4.70-6.01) | 4.73 (4.18-5.35) | 4.43 (3.90-5.02) |
| Quartile 4 | 11.98 (10.65-13.46) | 9.48 (8.42-10.68) | 7.77 (6.86-8.79) |
| *P* for trend | <0.001 | <0.001 | <0.001 |
| TyG-ABSI index |  |  |  |
| Per 1-SD increase | 1.90 (1.85-1.95) | 1.71 (1.67-1.76) | 1.58 (1.53-1.63) |
| Quartile 1 | Reference | Reference | Reference |
| Quartile 2 | 2.06 (1.86-2.28) | 1.88 (1.69-2.07) | 1.76 (1.59-1.95) |
| Quartile 3 | 3.42 (3.10-3.77) | 2.93 (2.65-3.23) | 2.58 (2.33-2.85) |
| Quartile 4 | 6.18 (5.62-6.81) | 4.78 (4.34-5.27) | 3.81 (3.44-4.22) |
| *P* for trend | <0.001 | <0.001 | <0.001 |
| TyG-WWI index |  |  |  |
| Per 1-SD increase | 2.06 (2.01-2.11) | 1.86 (1.82-1.91) | 1.74 (1.69-1.79) |
| Quartile 1 | Reference | Reference | Reference |
| Quartile 2 | 2.64 (2.34-2.97) | 2.42 (2.15-2.73) | 2.33 (2.06-2.62) |
| Quartile 3 | 4.63 (4.13-5.19) | 3.99 (3.56-4.47) | 3.65 (3.25-4.10) |
| Quartile 4 | 9.70 (8.70-10.82) | 7.43 (6.65-8.30) | 6.07 (5.41-6.82) |
| *P* for trend | <0.001 | <0.001 | <0.001 |

Model 1 were adjusted for age and sex.

Model 2 were adjusted for age, sex, ethnicity, employed status, educational levels, Townsend deprivation index, family income, physical activity, smoking status, alcoholic drinking, sleeping duration, and health diet score.

Model 3 were adjusted for age, sex, ethnicity, employed status, educational levels, Townsend deprivation index, family income, physical activity, smoking status, alcoholic drinking, sleeping duration, health diet score, total cholesterol, low density lipoprotein cholesterol, glycated hemoglobin, systolic/diastolic blood pressure, use of antihypertensive, antihyperglycemic, lipid-lowering medications, and self-reported history of hypertension, diabetes, and hyperlipidemia.

*Abbreviations*: HR hazard ratio, CI confidence interval, SD standard deviation; TyG triglycerides- glucose index, BMI body mass index, WC waist circumference, WHtR waist-to-height ratio; BRI body roundness index; ABSI a body shape index; WWI weight-adjusted-waist index.

**Figure S1** Subgroup analysis by sex for the associations between TyG-related indices and risk of incident MASLD

Models were adjusted for age, ethnicity, employed status, educational levels, Townsend deprivation index, family income, physical activity, smoking status, alcoholic drinking, sleeping duration, health diet score, total cholesterol, low density lipoprotein cholesterol, glycated hemoglobin, systolic/diastolic blood pressure, use of antihypertensive, antihyperglycemic, lipid-lowering medications, and self-reported history of hypertension, diabetes, and hyperlipidemia.

*Abbreviations*: HR hazard ratio, CI confidence interval, SD standard deviation; TyG triglycerides glucose index, BMI body mass index, WC waist circumference, WHtR waist-to-height ratio; BRI body roundness index; ABSI a body shape index; WWI weight-adjusted-waist index; MASLD metabolic dysfunction-associated steatotic liver disease.

**Figure S2** Subgroup analysis by age for the associations between TyG-related indices and risk of incident MASLD

Models were adjusted for sex, ethnicity, employed status, educational levels, Townsend deprivation index, family income, physical activity, smoking status, alcoholic drinking, sleeping duration, health diet score, total cholesterol, low density lipoprotein cholesterol, glycated hemoglobin, systolic/diastolic blood pressure, use of antihypertensive, antihyperglycemic, lipid-lowering medications, and self-reported history of hypertension, diabetes, and hyperlipidemia.

*Abbreviations*: HR hazard ratio, CI confidence interval, SD standard deviation; TyG triglycerides glucose index, BMI body mass index, WC waist circumference, WHtR waist-to-height ratio; BRI body roundness index; ABSI a body shape index; WWI weight-adjusted-waist index; MASLD metabolic dysfunction-associated steatotic liver disease.

**Figure S3** Subgroup analysis by smoking status for the associations between TyG-related indices and risk of incident MASLD

Models were adjusted for age, sex, ethnicity, employed status, educational levels, Townsend deprivation index, family income, physical activity, alcoholic drinking, sleeping duration, health diet score, total cholesterol, low density lipoprotein cholesterol, glycated hemoglobin, systolic/diastolic blood pressure, use of antihypertensive, antihyperglycemic, lipid-lowering medications, and self-reported history of hypertension, diabetes, and hyperlipidemia.

*Abbreviations*: HR hazard ratio, CI confidence interval, SD standard deviation; TyG triglycerides glucose index, BMI body mass index, WC waist circumference, WHtR waist-to-height ratio; BRI body roundness index; ABSI a body shape index; WWI weight-adjusted-waist index; MASLD metabolic dysfunction-associated steatotic liver disease.

**Figure S4** Subgroup analysis by alcoholic drinking for the associations between TyG-related indices and risk of incident MASLD

Models were adjusted for age, sex, ethnicity, employed status, educational levels, Townsend deprivation index, family income, physical activity, smoking status, sleeping duration, health diet score, total cholesterol, low density lipoprotein cholesterol, glycated hemoglobin, systolic/diastolic blood pressure, use of antihypertensive, antihyperglycemic, lipid-lowering medications, and self-reported history of hypertension, diabetes, and hyperlipidemia.

*Abbreviations*: HR hazard ratio, CI confidence interval, SD standard deviation; TyG triglycerides glucose index, BMI body mass index, WC waist circumference, WHtR waist-to-height ratio; BRI body roundness index; ABSI a body shape index; WWI weight-adjusted-waist index; MASLD metabolic dysfunction-associated steatotic liver disease.

**Figure S5** Subgroup analysis by self-reported hypertension for the associations between TyG-related indices and risk of incident MASLD

Models were adjusted for age, sex, ethnicity, employed status, educational levels, Townsend deprivation index, family income, physical activity, smoking status, alcoholic drinking, sleeping duration, health diet score, total cholesterol, low density lipoprotein cholesterol, glycated hemoglobin, systolic/diastolic blood pressure, use of antihypertensive, antihyperglycemic, lipid-lowering medications, and self-reported history of diabetes and hyperlipidemia.

*Abbreviations*: HR hazard ratio, CI confidence interval, SD standard deviation; TyG triglycerides glucose index, BMI body mass index, WC waist circumference, WHtR waist-to-height ratio; BRI body roundness index; ABSI a body shape index; WWI weight-adjusted-waist index; MASLD metabolic dysfunction-associated steatotic liver disease.

**Figure S6** Subgroup analysis by self-reported diabetes for the associations between TyG-related indices and risk of incident MASLD

Models were adjusted for age, sex, ethnicity, employed status, educational levels, Townsend deprivation index, family income, physical activity, smoking status, alcoholic drinking, sleeping duration, health diet score, total cholesterol, low density lipoprotein cholesterol, glycated hemoglobin, systolic/diastolic blood pressure, use of antihypertensive, antihyperglycemic, lipid-lowering medications, and self-reported history of hypertension and hyperlipidemia.

*Abbreviations*: HR hazard ratio, CI confidence interval, SD standard deviation; TyG triglycerides glucose index, BMI body mass index, WC waist circumference, WHtR waist-to-height ratio; BRI body roundness index; ABSI a body shape index; WWI weight-adjusted-waist index; MASLD metabolic dysfunction-associated steatotic liver disease.

**Figure S7** Subgroup analysis by self-reported hyperlipidemia for the associations between TyG-related indices and risk of incident MASLD

Models were adjusted for age, sex, ethnicity, employed status, educational levels, Townsend deprivation index, family income, physical activity, smoking status, alcoholic drinking, sleeping duration, health diet score, total cholesterol, low density lipoprotein cholesterol, glycated hemoglobin, systolic/diastolic blood pressure, use of antihypertensive, antihyperglycemic, lipid-lowering medications, and self-reported history of hypertension and diabetes.

*Abbreviations*: HR hazard ratio, CI confidence interval, SD standard deviation; TyG triglycerides glucose index, BMI body mass index, WC waist circumference, WHtR waist-to-height ratio; BRI body roundness index; ABSI a body shape index; WWI weight-adjusted-waist index; MASLD metabolic dysfunction-associated steatotic liver disease.
